# Human Tumor Targeted Cytotoxic Mast Cells for Cancer Immunotherapy

**DOI:** 10.3389/fonc.2022.871390

**Published:** 2022-04-22

**Authors:** Mohammad Fereydouni, Elnaz Ahani, Parth Desai, Mona Motaghed, Anthony Dellinger, Dean D. Metcalfe, Yuzhi Yin, Sung Hyun Lee, Tal Kafri, Aadra P. Bhatt, Kristen Dellinger, Christopher L. Kepley

**Affiliations:** ^1^ Department of Nanoscience, Joint School of Nanoscience and Nanoengineering, University of North Carolina at Greensboro, Greensboro, NC, United States; ^2^ Department of Nanoengineering, Joint School of Nanoscience and Nanoengineering, North Carolina Agricultural and Technical (AT) State University, Greensboro, NC, United States; ^3^ Laboratory of Allergic Diseases, National Institute of Allergy and Infectious Diseases, National Institutes of Health, Bethesda, MD, United States; ^4^ Gene Therapy Center and Department of Microbiology and Immunology, University of North Carolina at Chapel Hill, Chapel Hill, NC, United States; ^5^ Lineberger Comprehensive Cancer Center, and the Center for Gastrointestinal Biology and Disease, University of North Carolina at Chapel Hill, Chapel Hill, NC, United States; ^6^ Department of Molecular and Cellular Sciences, Liberty University College of Osteopathic Medicine, Lynchburg, VA, United States

**Keywords:** breast cancer, mast cell, IgE, FcεRI, TNF-α, AllergoOncology, adoptive cellular transfer, cell-based cancer immunotherapy

## Abstract

The diversity of autologous cells being used and investigated for cancer therapy continues to increase. Mast cells (MCs) are tissue cells that contain a unique set of anti-cancer mediators and are found in and around tumors. We sought to exploit the anti-tumor mediators in MC granules to selectively target them to tumor cells using tumor specific immunoglobin E (IgE) and controllably trigger release of anti-tumor mediators upon tumor cell engagement. We used a human HER2/*neu*-specific IgE to arm human MCs through the high affinity IgE receptor (FcεRI). The ability of MCs to bind to and induce apoptosis of HER2/*neu*-positive cancer cells *in vitro* and *in vivo* was assessed. The interactions between MCs and cancer cells were investigated in real time using confocal microscopy. The mechanism of action using cytotoxic MCs was examined using gene array profiling. Genetically manipulating autologous MC to assess the effects of MC-specific mediators have on apoptosis of tumor cells was developed using siRNA. We found that HER2/*neu* tumor-specific IgE-sensitized MCs bound, penetrated, and killed HER2/*neu*-positive tumor masses *in vitro*. Tunneling nanotubes formed between MCs and tumor cells are described that parallel tumor cell apoptosis. In solid tumor, human breast cancer (BC) xenograft mouse models, infusion of HER2/*neu* IgE-sensitized human MCs co-localized to BC cells, decreased tumor burden, and prolonged overall survival without indications of toxicity. Gene microarray of tumor cells suggests a dependence on TNF and TGFβ signaling pathways leading to apoptosis. Knocking down MC-released tryptase did not affect apoptosis of cancer cells. These studies suggest MCs can be polarized from Type I hypersensitivity-mediating cells to cytotoxic cells that selectively target tumor cells and specifically triggered to release anti-tumor mediators. A strategy to investigate which MC mediators are responsible for the observed tumor killing is described so that rational decisions can be made in the future when selecting which mediators to target for deletion or those that could further polarize them to cytotoxic MC by adding other known anti-tumor agents. Using autologous human MC may provide further options for cancer therapeutics that offers a unique anti-cancer mechanism of action using tumor targeted IgE’s.

## Introduction

The tumor microenvironment is a complex mixture of resident stromal cells, infiltrating hematopoietic cells, a heterogeneous population of cancer cells, and tissue mast cells (MCs) arising from hematopoetic progenitors. Mast cells are unique immune cells that secrete a diverse array of biologically active compounds that can stimulate, modulate, or suppress an immune response. The role of MCs in most forms of cancer has been investigated and depending on the model and experimental design, they appear to exhibit either a pro- or anti-tumorigenic role (1–4). This is due in part to observational/correlative studies performed in humans and contradictory roles concluded from rodent MC *in vivo* models given the known differences in human vs. murine MCs (5, 6). Increased histamine decarboxylase gene expression, responsible for MC histamine expression, is associated with better relapse-free and overall survival in breast cancer (BC) patients (7). Histamine thus reduces tumor growth and increases apoptosis of cancer cells *in vivo (* 8). Further, human MC granules contain stored, releasable (through FcϵRI) tumor necrosis factor alpha (TNF-α) (9). In addition, we and others have demonstrated that human MC release significant amounts (2,500-4,000 pg/ml from 10^5^ cells) of granulocyte-macrophage colony-stimulating factor (GM-CSF) upon FcϵRI exposure (10). This is relevant as both TNF-α and GM-CSF suppress tumor cell proliferation, induce tumor regression, enhance anti-tumor co-therapies, and have been investigated in over 50 clinical trials (11, 12).

Biologics are being investigated for cancer immunotherapy and the number and variety of FDA-approved, humanized Abs to treat various cancers continues to grow (13). While all are of the human IgG class, IgE has several potential advantages over IgG which has led to the development of several tumor targeted, humanized or chimeric IgE’s with distinct anti-tumor immune responses compared to IgG’s (14, 15). While the contribution of MCs in studies examining the anti-tumor effects of IgE have been largely overlooked, it is highly plausible that the IgE Fc binding to MC-FcεRI mediates the anti-tumor effects given the affinity of this interaction (16), the juxtaposition of MCs to tumor cells (17), and the anti-tumor mediators within MCs that are released by the interaction of IgE with tumor antigens (10, 18).

These observations form the rationale for these studies: that MCs play a beneficial and exploitable role in cancer microenvironments. This potential anti-tumor effect from the addition of *ex vivo* derived, tumor-targeted, IgE-sensitized MCs into tumor proximity has not been investigated. We thus hypothesized that MCs could be polarized to direct their anti-tumor mediators within the local tumor habitat with a targeted release dependent on a controllable triggering system activated only when encountering tumor-specific antigens. To explore this concept, we used both *in vitro* and *in vivo* models targeting human epidermal growth factor receptor 2 (HER2/*neu*) which is already a target for several IgG-based immunologics for cancer therapy (19). We demonstrate that autologous human MCs have potent anti-tumor properties that can be controllably released upon tumor cells engagement. The MC-induced apoptosis was dependent on tumor IgE, occurred *in vitro* and *in vivo*, and revealed a unique array of tumor genes affected by MCs incubation. MCs could also be genetically manipulated to potentially decrease pro-tumor or toxic mediators and conversely to increase anti-tumor mediators. We present evidence that autologous MCs can be polarized to direct their hyper-inflammatory, anti-tumor function as a novel strategy for cancer cell immunotherapy.

## Materials and Methods

### Kinetics of Tumor Antigen-Induced MC Mediator Release

Human MCs cultured from peripheral blood (BDMC) obtained from healthy adult volunteers as described after informed consent was obtained under a protocol approved by the Institutional Review Board of the National Institute of Allergy and Infectious Diseases (2009-I-0049) (20). When cultures reached at least six weeks of age they were sensitized with or without 0.1 μg/ml human anti-HER2/*neu* IgE (from Absolute Antibody, Boston, MA) as described previously with adipose-derived MCs (ADMC) and skin derived MCs (10). After 1 h, cells were washed and filtered (40 μm) HER2/*neu*-positive BT474 (ATCC, Manassas, VA) or SK-BR-3 (SL032; Genecopoeia, Inc, Rockville, MD) cells were added at the indicated number and mediator release and apoptosis measured as described (21). All experiments were performed in duplicate from three separate donors and significant differences (*p*<0.05) were determined using the Student *t-*test.

### Production of Luciferase-Transduced Cancer Cell Lines

BT474 cells were transduced with the lentiviral vector pTK1261 (CMV-Luc-GFP/BSD) containing Firefly luciferase, and the fusion GFP/Blastcidine selection marker gene. Cells were washed with media containing Blasticidin S (20 μg/ml) four times to remove non-infected cells. The incorporation of the Firefly gene was confirmed by a luciferase assay that resulted the light intensity of 3,545 relative light units (RLU)/μg, compared to 13 RLU/μg of control, non-infected cells revealing approximately 100% of BT474-1261 cells expressed luciferase protein (**Supplementary Figure 3**). Luciferase/GFP dual-labeled SK-BR-3 cancer cell line was purchased from GeneCopoeia, Inc. Rockville, MD.

### Time Lapse Confocal Microscopy

To assess the ability of anti-HER2/*neu* IgE sensitized MCs to induce cell death of HER2/*neu* expressing tumor cells, MCs (1.5 x 10^5^) were sensitized with 0.1 μg/ml of anti-HER2/*neu* IgE or psIgE for 2 h. Filtered (40 μm) BT474-1261 or SK-BR-3 breast cancer cells (5 x 10^4^) on coverslips were labeled with MitoTracker™ Red (1 μM, ThermoFisher Scientific) for one hour. The washed MCs were labeled with CellTracker™ Deep Red (1 μM) for one hour, washed, and added to the cancer cells in medium containing 7 μM of CellEvent™ Caspase 3/7 Green (to detect activated caspase-3/7 in apoptotic cells; Invitrogen) for 1 h according to the manufacturers protocol. Time-lapse photography was recorded over a period of up to four days in a live cell chamber as described (10).

### Scanning Electron Microscopy

Cocultures of ADMC and filtered BT474-1261 were treated as above, washed, and fixed with 2.5% glutaraldehyde and 4% formaldehyde in PBS for 2 h. Following three rinses with distilled water, the samples were dehydrated through a gradient series of ethanol (50%-100%) and further dehydrated using a critical point dryer and hexamethyldisilazane (HMDS) to preserve the cell surface protrusions. Specimens were set on stubs by conductive double-sided carbon tape and covered by 10 nm thick gold-palladium by a sputter coater (Leica Microsystems, IL, USA). Cells were examined using a field emission scanning electron microscope (Zeiss Auriga FIBFESEM, Zeiss, NY, USA) at 4 kV.

### Maximum Tolerated Dose, *In Vivo* Imaging and Efficacy of ADMC

No studies have examined the maximum tolerated dose (MTD) of human MCs *in vivo*. To assess this MTD, female Nu/Nu mice weighing 20–28 g between 8 and 16 weeks of age were bred and maintained in a pathogen-free animal facility. Mice (6/group) were injected i.v. with or without the indicated number of cells in PBS with an insulin syringe with a 28 Ga needle and following isoflurane anesthesia. Serums were collected before and after injection for analysis and weights taken daily for comparison between control and ADMC injected mice.

For *in vivo* imaging female Nu/Nu mice were implanted with 60 day 17-β-estradiol pellets (Innovative Research of America) subcutaneously 3 days before implantation of luciferase-transduced BT474-1261 tumors. Tumors were implanted as single cell suspensions (2 x 10^6^ cells) into the subcutaneous sacral region or into the inguinal mammary fat pads. When tumors reached ≥200 mm^3^ mice were injected intratumorally or intravenously with IgE-sensitized (0.1 μg/ml for 2 h), CellBrite 680-labelled ADMC using the indicated number of cells in PBS. For *in vivo* optical imaging, mice were anesthetized with inhalation of isoflurane mixed with oxygen, and maintained under anesthesia during imaging. The CIVIS-Spectrum optical imaging system with excitation filter and emission filter at 675nm, 720 nm, respectively, was used to conduct fluorescence imaging before and after the injection of ADMC and *in vivo* optical imaging was taken at different time points. After each imaging session, animals were recovered from anesthesia and placed in the normal housing cage between imaging time points. At the end of the last imaging time point, animals were euthanized, and tumor tissue was collected for histology and flow cytometry analysis. For 2D optical image analysis, regions of interest were drawn on the tumor region of each animal, and the total radiant efficiency (excitation normalized fluorescence signal) was measured using Living Imaging software (PerkinElmer, Inc.), and plotted against imaging time points. In addition, to colocalize the fluorescence signal in tumor, 3D fluorescence imaging was also conducted using the IVIS-Spectrum imaging system, followed immediately by a CT imaging using an *in vivo* microCT imaging system (Quantum-GX, PerkinElmer, Inc.) with animals kept under anesthesia in a multi-modality imaging shuttle. The 3D optical images and CT images were co-registered to localize the fluorescence signal in tumor regions.

To assess the anti-tumor effects of ADMC in tumor bearing mice, tumors were generated as above. When tumors reached ∽200 mm^3^, mice (4/group) mice were injected i.t. with PBS or HER2/*neu* sensitized ADMC (1x10^6^) at day 0 and tumor volume and mean survival were assessed. Primary tumor volumes (TV) were calculated according to the National Cancer Institute (NCI; Bethesda, MD) protocol [TV= (length x width^2^)/2], where “length” and “width” are the long and short diameters of the tumor mass in millimeters. The significance of the differences in tumor volume was determined using the two-tailed Student’s *t-*test and survival by the non-parametric Peto-Peto-Wilcoxon Log-Rank test. Samples of blood were taken for MC mediator analysis and toxicological indicators (22). Tumors were removed to assess the presence of MCs by H&E and immunohistochemistry with anti-tryptase Abs (clone G3; a gift from Lawrence Schwartz, VCU Health Systems) that do not cross react with mouse MC tryptases (23).

### Gene Expression Profiling of mRNA

RNA sequencing was performed on BT474-1261 cells *in vitro* and BT474-1261 tumors from *in vivo* experiments. Anti-HER2/*neu* IgE, PBS, or psIgE sensitized ADMC (2 x 10^6^) were incubated with filtered BT474-1261 cells (1 x 10^6^) or tumors (>200 mm^3^) at days one and four. Preparations of the ADMC used for these experiments were from male donors and the BT474-1261 cells are female so that the up- and down-regulation of RNA’s is focused just on the tumor cells through gating of XY vs XX chromosomes. Total RNA was extracted using Qiagen RNeasy Plus Universal mini kit following manufacturer’s instructions (Qiagen, Hilden, Germany). Extracted RNA samples were quantified using Qubit 2.0 Fluorometer (Life Technologies, Carlsbad, CA, USA) and RNA integrity was checked using Agilent TapeStation 4200 (Agilent Technologies, Palo Alto, CA, USA).

### Library Preparation With Poly-A Selection and HiSeq Sequencing

RNA sequencing libraries were prepared using the NEBNext Ultra II RNA Library Prep Kit for Illumina following manufacturer’s instructions (NEB, Ipswich, MA, USA). Briefly, mRNAs were first enriched with Oligo(dT) beads. Enriched mRNAs were fragmented for 15 minutes at 94°C. First strand and second strand cDNAs were subsequently synthesized. cDNA fragments were end repaired and adenylated at 3’ends, and universal adapters were ligated to cDNA fragments, followed by index addition and library enrichment by limited-cycle PCR. The sequencing libraries were validated on the Agilent TapeStation (Agilent Technologies, Palo Alto, CA, USA), and quantified by using Qubit 2.0 Fluorometer (Invitrogen, Carlsbad, CA) as well as by quantitative PCR (KAPA Biosystems, Wilmington, MA, USA). The sequencing libraries were clustered on 1 lane of a flowcell. After clustering, the flowcell was loaded on the Illumina HiSeq instrument according to manufacturer’s instructions. The samples were then sequenced using a 2x150bp Paired End (PE) configuration. Image analysis and base calling were conducted by the HiSeq Control Software (HCS). Raw sequence data generated from Illumina HiSeq was converted into fastq files and de-multiplexed using Illumina’s bcl2fastq 2.17 software. One mismatch was allowed for index sequence identification.

For data analysis, sequence reads were trimmed to remove possible adapter sequences and nucleotides with poor quality using Trimmomatic (v.0.36). The trimmed reads were mapped to the *Homo sapiens* reference genome available on ENSEMBL using the STAR aligner v.2.5.2b. The STAR aligner is a splice aligner that detects splice junctions and incorporates them to help align the entire read sequences. BAM files were generated as a result of this step. Unique gene hit counts were calculated by using feature Counts from the Subread package v.1.5.2. Only unique reads that fell within exon regions were counted. After extraction of gene hit counts, the gene hit counts table was used for downstream differential expression analysis. Using DESeq2, a comparison of gene expression between the groups of samples was performed. The Wald test was used to generate p-values and Log2 fold changes. Genes with adjusted p-values < 0.05 and absolute log2 fold changes > 1 were called as differentially expressed genes for each comparison. A gene ontology analysis was performed on the statistically significant set of genes by implementing the software GeneSCF. The goa_human (GO) list was used to cluster the set of genes based on their biological process and determine their statistical significance. A PCA analysis was performed using the “plotPCA” function within the DESeq2 R package. The plot shows the samples in a 2D plane spanned by their first two principal components. The top genes, selected by the highest row variance, were used to generate the plot (Genewiz, South Plainfield, NJ).

### Construction of Lentiviral Particles and Transduction of ADMC and Adipose Stem Cells

The 293T HEK cell line expanded in 12 x 15 cm dishes until 80-90% confluency and transfected by plasmids containing Green Fluorescent Protein (GFP) (Verma Lab, Salk Institute for Biological Studies) and three additional plasmids (pMDL, pRev and pVSVG) (Invitrogen cat. no. K4975-00) for viral packaging. To make the plasmid mix, 270 μg of transfer vector, 176 μg of pMDL (Gag/Pol), 95 μg of pVSVG (vesicular stomatitis virus glycoprotein) and 68 μg of pREV, were added to 13.5 ml of 0.25M CaCl_2_ + 13.5 ml 2x BBS solution. The transfection mixture was spread in drops to each plate (2.25 ml/plate) and incubated overnight. Afterward, the media was removed and 15 ml of fresh DMEM + 2% FBS was added to each dish and incubated overnight. The next day, the supernatants were collected, stored at 4°C, and 15 ml fresh media added to the cells. The second harvest of supernatant was collected the next day. To concentrate the viral particles ultracentrifugation was applied at 70,000 *x g* for 2 h at 4°C, which yielded a final concentration of 10^9^ particles per ml. MCs were seeded in a 24 well plate (10^5^ cells/well) and different amounts of virus (5, 10, 20 and 30 μl) were used to infect the cells.

## Results

### Peripheral Blood-Derived Human MCs Become Activated Through FcεRI Upon HER2/*neu* -Positive Breast Cancer Cell Binding

**Figure 1:**
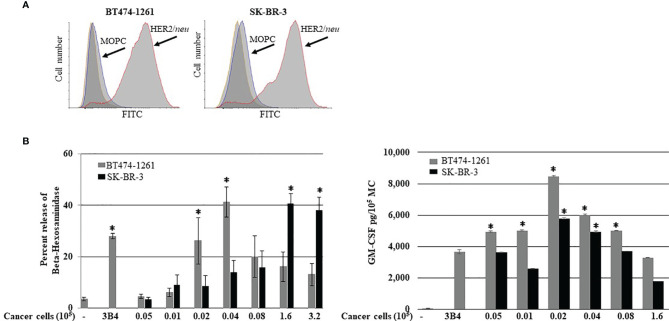
Breast cancer cell-induced BDMC mediator release. **(A)** Expression of HER2/*neu* on BT474-1261 or SK-BR-3 cells using FACS. BDMC were sensitized with 1 μg/ml anti-HER/*neu* IgE washed, and incubated with filtered BT474-1261 or SK-BR-3 cells and degranulation (**B**; left) and cytokine release (**B**; right) assessed. Data are from a single experiment representative of experiments performed on cells derived from three separate donors. Error bars represent ±SD. *p<0.05 compared with non-IgE (spontaneous) release.

It has been reported that HER2/*neu* IgE-sensitized ADMC release anti-tumor mediators upon FcεRI crosslinking when challenged with HER2/*neu*-positive BC cells (SK-BR-3 and BT474-1261) (10). Another autologous source of human MCs that could be utilized for potential cellular-based cancer immunotherapy is the generation of human MCs from precursors in peripheral blood (20). Thus, the ability of BDMC sensitized with the anti-HER2/*neu* IgE to degranulate in the presence of BC cells was investigated. Both SK-BR-3 and BT474-1261 expressed high amounts of HER2/*neu* (**Figure 1A**). The BDMC exhibited significant (*p*<0.05) degranulation and cytokine production through FcεRI when co-incubated with BDMC sensitized with anti-HER2/*neu* IgE (**Figure 1B**). Thus, ADMC, skin-derived, and BDMC respond similarly to BC-induced mediator release (10).

### Scanning Electron Microscopy of ADMC Interactions With BT474-1261 Cells

**Figure 2:**
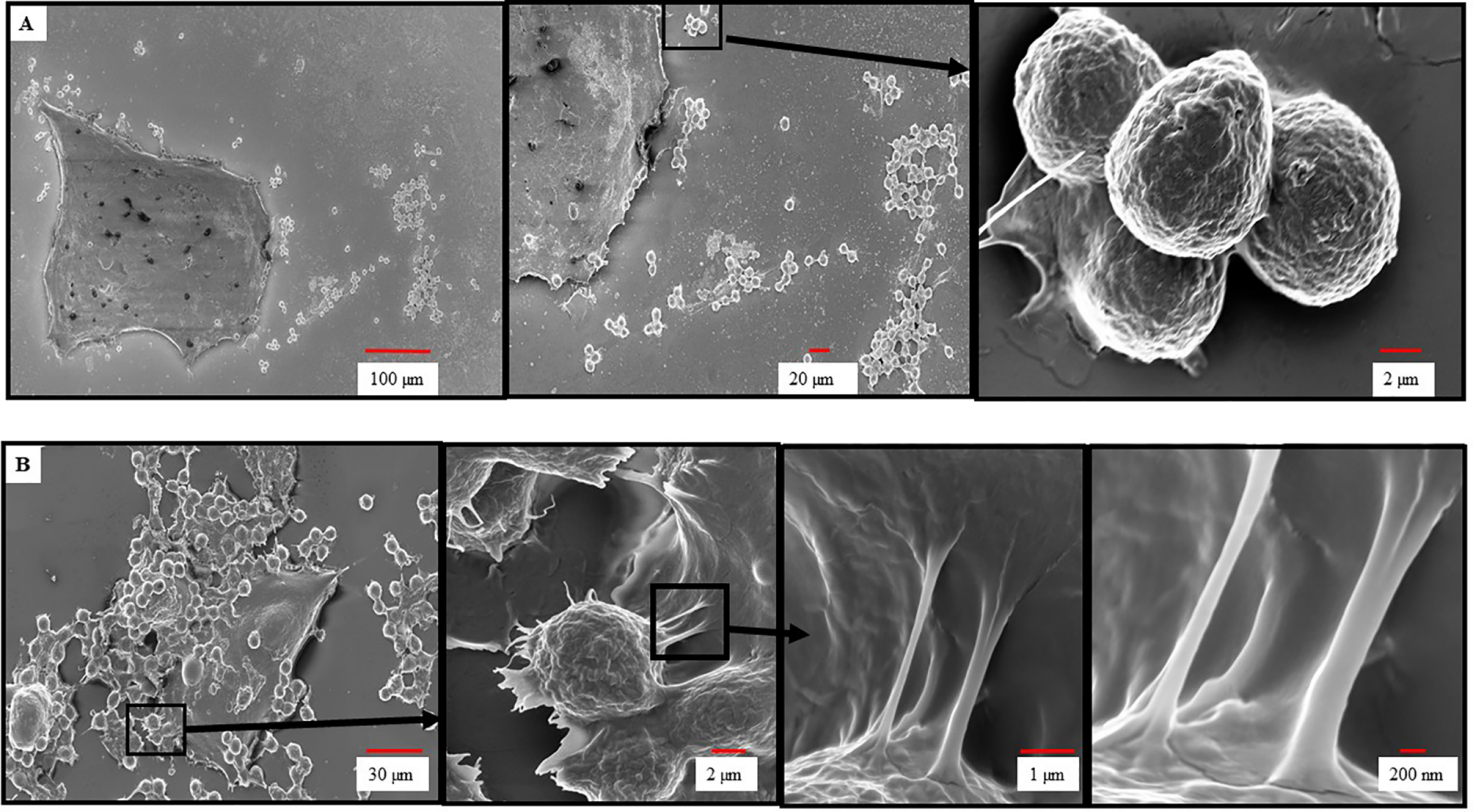
Scanning electron microscopy of ADMC and BC cell interactions. ADMC (1.5×10^5^) were sensitized with 1μg/ml of non-specific IgE **(A)** or anti-HER2/*neu* IgE **(B)** for 1 h, washed, and added to HER2/*neu*+ BT474-1261 cells (about 60% confluence) and incubated for 24 h at 37°C. Cells were collected, supernatants removed for β-hexosaminidase release, and samples fixed for SEM as described above. Arrows denote magnified areas.

To further investigate the dependence of HER2/*neu* IgE in mediating the binding of MCs to BC cells we performed SEM to examine the nature of this interaction. As seen in **Figure 2A**, ADMC sensitized with non-specific IgE did not bind to the BT474-1261 and displayed smooth membranes with few membrane protrusions as determined by SEM. In contrast, the HER2/*neu* IgE-sensitized ADMC bound to HER2/*neu*-positive BT474-1261 cells (**Figure 2B**). Analysis of the specific interaction between the ADMC and BT474-1261 cells indicated the cells were in the process of degranulation (up to 50%; **Figure 1B**) with the appearance of ruffling, lamellipodia-like membrane ridges, and cellular protrusions. Higher magnifications (≤ 2 μm) indicate these protrusions attach to the BC cells through heterocellular, tunneling nanotube (TnT)-like structures. We hypothesized that TnT transport MC-specific molecules into the cancer cells. Further investigation is required to elucidate the role of TnT in cancer cell apoptosis accurately. These results demonstrate that HER2/*neu* IgE regulates MC binding and reveals that this process involves the formation of cellular protrusions between FcεRI-crosslinked MCs with tumor cells. We are currently examining the significance of these TnT to determine if they represent a structure by which mediators and/or organelles could be exchanged.

### Time-Lapse Confocal Microscopy of HER2/*neu* IgE Sensitized ADMC Inducing Apoptosis of BT474-1261 Cells

**Figure 3:**
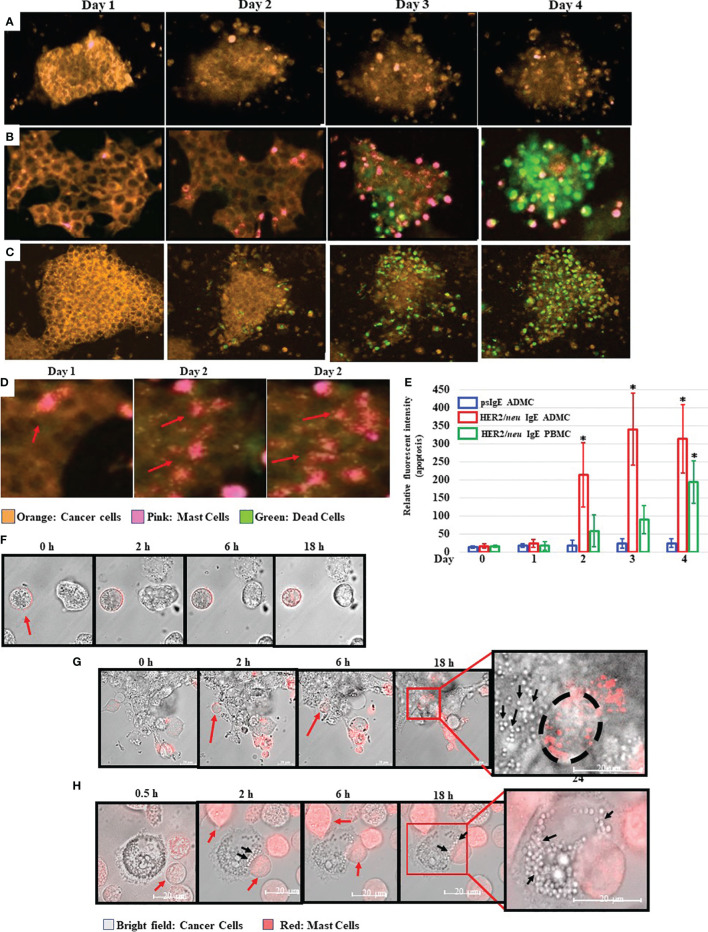
Mast cells bind to, penetrate, degranulate, and induce FcεRI-dependent apoptosis of BC cells. CellTracker™-Deep Red labelled ADMC **(A, B)** or BDMC **(C)** (7.5x10^4^) were sensitized with 1 μg/ml non-specific IgE **(A)** or anti-HER2/*neu* IgE **(B, C)**, washed, and incubated with MitoTracker™-Red HER2/*neu*+ BT474-1261 in culture medium containing a fluorescent apoptosis detecting dye (CellEvent Caspase 3/7; 1:100). Cells were monitored in a live cell chamber attached to a confocal microscope for up to 4 days. **(D)**. The amount of fluorescent intensity of the apoptosis dye was quantified using Image J of the average of ten fields of view at 63x magnification (±SD, *P < 0.05) **(E)**. Higher magnifications of several degranulating MCs is shown to illustrate the release and spreading of MC granules upon cancer cell HER2/*neu*-induced activation of FcεRI. The red arrows indicate spreading MC granules within the BT474-1261 cancer cell mass. **(F–H)**. MitoTracker™-Red labelled MCs (7.5x10^4^) were sensitized with 1 μg/ml non-specific IgE **(F)** or anti-HER2/*neu* IgE **(G, H)**, washed, and incubated with HER2/*neu*+ BT474-1261 **(F, G)** or SK-BR-3 **(H)** cells and monitored in a live cell chamber for the indicated times. The red arrows indicate the dye-labelled ADMC, black arrows indicate cancer cell apoptotic bodies, and black circle indicates the outline of the ADMC within the tumor cell mass. Experiments are representative of 3 different donors.

The ability of MCs to induce BC cell death *in vitro* was next investigated. As expected, the BT474-1261 cell killing was dependent on the anti-HER2/*neu* IgE, as non-specific IgE-sensitized ADMC did not bind to or induce significant BT474-1261 apoptosis (**Figure 3A**) compared to those ADMC sensitized with anti-HER2/*neu* IgE (**Figure 3B**). Strikingly, the anti-HER2/*neu* IgE-sensitized ADMC appeared to penetrate and migrate through the cell tumor masses as seen in the time-lapse video using ADMC (**Supplementary Video 1**). The binding of anti-HER2/*neu* IgE-sensitized BDMC to BT474-1261 cells also induced apoptosis of the BC cells (**Figure 3C** and **Supplementary Video 2**). Higher magnifications demonstrated the anti-HER2/*neu* IgE-sensitized ADMC degranulated upon binding and internalization into HER2/*neu* cancer cells as seen by the increase in MC granules and translucent apoptotic bodies within the tumor cells (**Figure 3D**). Quantification of the apoptotic cells from psIgE ADMC challenged BC cells and HER2/*neu* IgE sensitized ADMC and BDMC challenged cells is shown in **Figure 3E**. To further examine these early (<24 h) interactions, we found that non-labelled BT474-1261 cells challenged with non-specific IgE-sensitized ADMC did not bind to or induce BT474-1261 apoptosis (**Figure 3F**). The anti-HER2/*neu* IgE-sensitized ADMC were observed to migrate and be internalized where they appeared to degranulate within the BT474-1261 (**Figure 3G**) and SK-BR-3 (**Figure 3H**) cells. The formation of apoptotic bodies within the cancer cells was evident initially at the point of MC:tumor cell binding which covered the whole inner cell after ∽24 h. Further, the formation of apoptotic bodies appeared at the contact points between the MC and cancer cells, suggesting a bi-directional release of anti-tumor mediators following FcεRI activation (**Figure 3H**). These experiments indicate anti-HER2/*neu*-sensitized MCs bind to, penetrate, degranulate, and induce apoptosis of HER2/*neu*-positive cancer cells and cell masses.

### Mast Cells Induce Cancer Cell Apoptosis Through TNF-α and Apoptosis Related Genes

**Figure 4:**
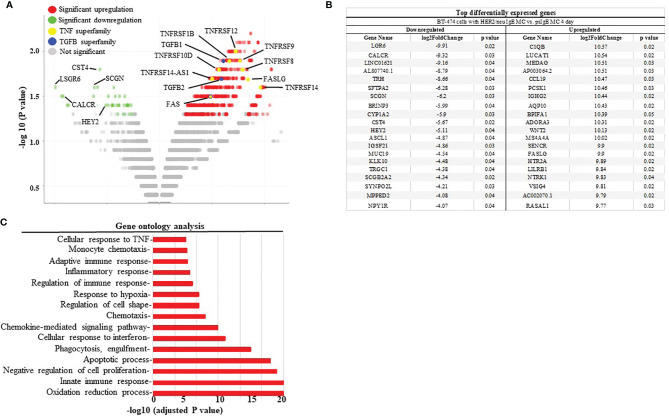
Differential gene expression analysis BT474-1261 cells treated of HER2/*neu* IgE-sensitized vs. non-specific IgE-sensitized ADMC. **(A)** Volcano plot of global transcriptional changes of BT474-1261 cells treated with ADMC sensitized HER2/*neu* IgE vs ADMC sensitized psIgE. Each data point in the scatter plot represents a gene. The ADMC were from male donors so that they could be distinguished from BT474-1261 cells by Y-chromosome gene mapping. Red and green indicate significant (P < 0.05) differences in control vs. treated cells. The log2 fold change of each gene is represented on the x-axis and the log10 of its adjusted p-value is on the y-axis. Genes with an adjusted p-value less than 0.05 and a log2 fold change greater than 1 are indicated by red dots (upregulated genes). Genes with an adjusted p-value less than 0.05 and a log2 fold change less than -1 are indicated by green dots (downregulated genes). **(B)** Top genes significantly affected by HER2/*neu* vs control ADMC. **(C)** Gene ontology analysis. Significantly (adjusted P-value <=0.05) differentially expressed genes were clustered by their gene ontology and the enrichment of gene ontology terms were tested using Fisher exact test (GeneSCF v1.1-p2).

To further assess the mechanisms of how MCs induce cancer cell death the up- or down-regulation of RNA was assessed in MCs sensitized with tumor-specific vs. non-specific IgE and BT474-1261 cells *in vitro*. As seen in **Figure 4A**, gene expression analysis of tumor cells challenged with tumor-IgE ADMC revealed a significant upregulation of several members of the TNF superfamily (TNFSF). This included the TNF-related apoptosis-inducing ligand (TRAIL/TNFRSF10), which further supports a role for FcϵRI-release of TNF-α from MCs that induces apoptosis of cancer cells (10). Additionally, significant upregulation of apoptotic genes such as TNF ligand superfamily members were observed which are targets for clinical development (24). Concomitantly, the FcεRI-induced activation of ADMC induced significant downregulation of several genes shown to be markedly upregulated in certain cancers and metastasis including cystatin 4 (CST4), secretagogin (SCGN), calcitonin receptor (CALCR), hairy and enhancer-of-split related with YRPW motif (HEY2), and the leucine-rich-repeat-containing G protein-coupled receptor 6 (LGR6) (25–30) (**Figure 4B**). Gene ontogeny analysis confirmed significant effects on the cellular response to TNF, apoptosis, and pro-inflammatory pathways (**Figure 4C**).

### HER2/*neu* IgE Sensitized ADMC Shrink HER2/*neu*-Positive Tumors *In Vivo*

**Figure 5:**
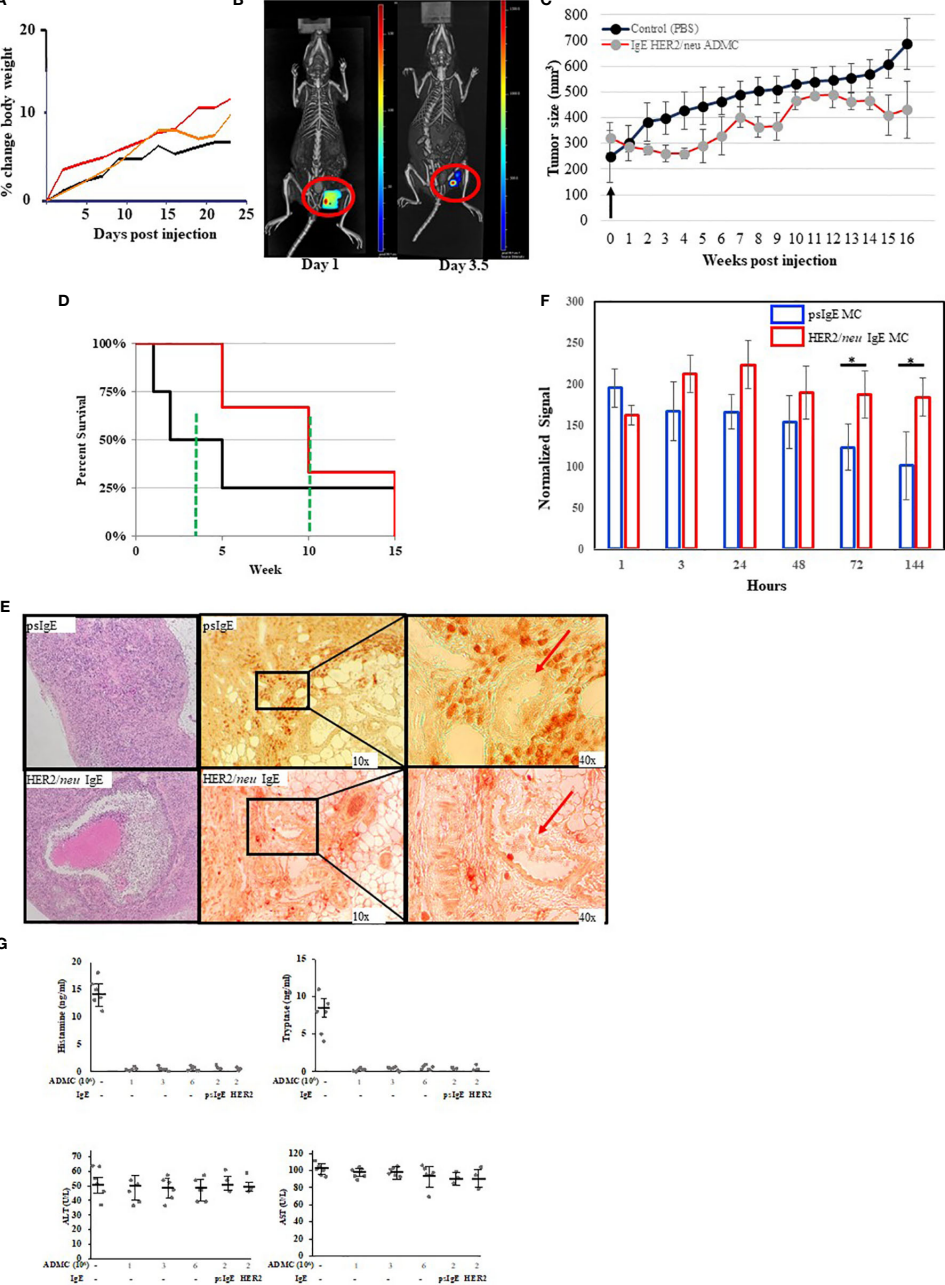
*In vivo* analysis of ADMC. **(A)** Maximum tolerated dose of ADMC *in vivo.* NU/NU mice (female; 8-week-old; 5 per group) injected i.v. with 1x10^6^ (black), 3x10^6^ (orange), or 6x10^6^ (red) ADMC and mouse weights analyzed over time shown. No significant loss in body weight was observed to indicate toxicity. **(B–D)**. Tumor binding and anti-tumor activity of ADMC *in vivo.* Tumors were prepared by injecting Nu/Nu mice with 2x10^6^ HER2/*neu*+ BT474-1261 cells. Mice were injected i.t. with 1x10^6^ HER2/*neu* **(B)** monitored over 7 days using 3D fluorescence imaging with an IVIS-Spectrum imaging system, followed immediately by a CT imaging using an *in vivo* microCT imaging system. **(C, D)** Antitumor activity of ADMC in HER2/*neu* positive human BC tumors in Nu/Nu immunocompromised mice. Tumors were prepared as in **(B)**. When tumors reached ∽200 mm^3^, mice (4/group) were injected i.t. with or without (PBS), HER2/*neu* or psIgE sensitized ADMC (2x10^6^) at day 0 and tumor volume **(C)** and mean survival **(D)** assessed. Points, mean tumor volume (mm^3^); bars, ±SD. The green line represents the mean survival for each group. **(E)** Tumors obtained from mice treated as above (day 4) were incubated with H&E (left), mouse anti-human tryptase (middle), or MOPC (**Supplementary Figure 2**) followed by peroxidase anti-mouse Abs. Red arrows are blood vessels. HER2/*neu* IgE-sensitized ADMC are evenly distributed inside the tumor tissue (bottom), while psIgE-sensitized ADMC are sparse inside the tumor tissue (top) and are mostly found in close vicinity of blood vessels, where they flow into the blood stream. **(F)** Quantification of ADMC numbers was assessed by monitoring the fluorescence of the CellBrite signal in the tumors *in vivo* over time. Data are represented as the mean ± s.e.m. of n = 3 technical replicates. *P < 0.05. **(G)** Cytotoxic human MCs do not induce systemic MC release or toxicity. NU/NU mice (no tumors) were injected i.v. with indicated numbers of ADMC. Serum was collected at 1 hour (histamine and tryptase) or after 8 weeks (ALT/AST) and assessed for each mediator using commercially available kits. In last 2 points comparing psIgE vs HER2/*neu* IgE sensitized ADMC injected mice had established (>200 mm^3^) HER2/*neu*-positive tumors as above. Spiked mouse serums were used as controls for histamine and tryptase. Data are represented as mean ± s.e.m. No statistical significance change in serum levels was observed using ANOVA between non-ADMC treated animals vs treated.

We next tested the anti-tumor activity of ADMC *in vivo* using a human BC cell xenograft model with BT474-1261. In preparation for *in vivo* studies, experiments were performed to assess the MTD of ADMC. As seen in **Figure 5A**, no toxicity was observed (as assessed by the expected normal >20% change in body weight). Additionally, no other overt signs of distress (e.g. anaphylaxis, death) were observed in mice using up to 6x10^6^ ADMC i.v. To assess the efficacy of ADMC as anti-tumor cells, luciferase-luciferase-expressing HER2/*neu+* BT474-1261 cells were implanted in immunocompromised mice then injected intratumorally (i.t.) with dye-labelled HER2/*neu* IgE-sensitized ADMC cells and visualized using a whole-body scanner. As seen in **Figure 5B** and **Supplementary Video 3** injecting tumors i.t. demonstrated that ADMC could be visualized and observed to shrink the tumor after 4 days. It is demonstrated for the first time that mice expressing HER2/*neu*+ tumors injected with HER2/*neu* IgE-sensitized ADMC have significantly reduced tumor size and increased survival rates (>30%) compared to those labelled with non-specific IgE (**Figures 5C, D**). Importantly, mice exhibited no change in behavior characteristic of anaphylaxis (**Supplementary Video 4**), respiratory rate or body temperature when injected with HER2/*neu* IgE-sensitized ADMC, consistent with the conclusion that IgE-activation does not induce systemic anaphylaxis. Tumors obtained at day 4 post injection stained with H&E (**Figure 5E**; left) showed clear differences with large areas where apoptotic cells and debris could be observed (**Supplementary Figure 1**) in the HER2/*neu* IgE MCs challenged animals that were not observed in the controls. Immunohistochemistry with anti-human tryptase (**Figure 5E**, right) or control Abs (**Supplementary Figure 2**) revealed HER2/*neu* IgE MCs were retained within tumors. Non-specific IgE sensitized MCs were sparsely found in the primary tumor area but instead were observed around blood vessels in the tumor which we hypothesize is how these cells exit the tumor. It was verified that the psIgE ADMC did not persist within the tumor as did the HER2/*neu* IgE sensitized ADMC as measured by MC signals (**Figure 5F**). Serum histamine and human tryptase were not detected following *in vivo* ADMC challenge and liver function as determined by ALT and AST levels were no different in ADMC challenged and non-challenged mice (**Figure 5G**). These experiments suggest that MCs have anti-tumor activity *in vivo* and promote a localized anti-tumor function in the tumor microenvironment and do not induce anaphylaxis or liver toxicity.

**Figure 6:**
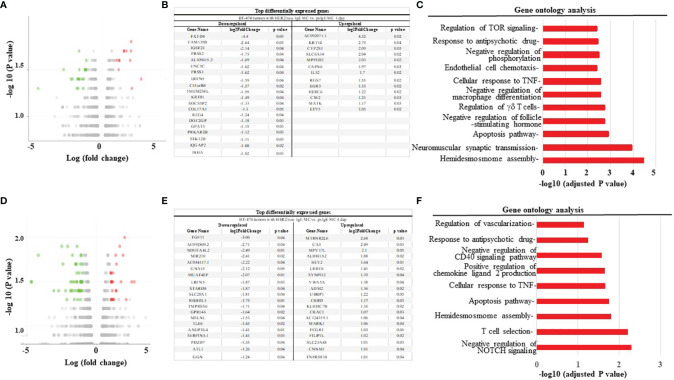
HER2/*neu*-sensitized ADMC induce apoptosis of HER2/*neu*-positive BT474-1261 tumors through a unique set of gene pathways. **(A, D)** Volcano plot of differentially expressed genes in tumors from mice engrafted with human female BT474-1261 cells. When tumors reached 200 mm^3^, 1 x 10^6^ HER2/*neu* or psIgE sensitized ADMC from a male human donor were injected intratumorally. Tumors were harvested after 1 **(A)** or 4 day **(D)** and scRNAseq was performed on the tumor samples. Adoptively transferred ADMC were distinguished from BT474-1261 tumors by Y-chromosome gene mapping. Red and green indicate significant (P < 0.05) differences in psIgE vs HER2/*neu* treated animals. Each data point in the scatter plot represents a gene. The log2 fold change of each gene is represented on the x-axis and the log10 of its p-value is on the y-axis. Genes with a p-value less than 0.05 and a log2 fold change greater than 1 are indicated by red dots (upregulated) and those with less than -1 are indicated by green dots (downregulated). The top genes significantly up or down regulated are shown at days 1 **(B)** and 4 **(E)**. Gene ontology analysis was performed on the statistically significant set of genes by implementing the software GeneSCF v.1.1-p2. The mgi GO list was used to cluster the set of genes based on their biological processes and determine their statistical significance. A list of genes clustered based on their gene ontologies was generated for tumors treated with HER2/*neu* IgE-sensitized vs. psIgE sensitized ADMC at day 1 **(C)** and 4 **(F)**.

We next used the xenograft tumor model to examine the anti-tumor mechanisms of MCs. The mRNA from tumors obtained from mice treated with HER2/*neu* IgE sensitized MCs was compared to those tumors obtained mice treated with psIgE-sensitized MCs. Overall, there was a downregulation of pro-tumor intermediates and pathways and an upregulation of those pathways involved in apoptosis and tumor inhibition. For example, at one day post MC infusion, gene expression analysis (**Figures 6A–C**) revealed a significant upregulation of genes associated with therapeutic benefits of trastuzumab [e.g. MATK (31)], tumor suppressors [RGS7 (32), MPPED2 (33)], and tumor cell apoptosis [IL-32 (34)]. Similarly, at day 4 MC-treated tumors had significant upregulation of inhibitors of angiogenesis [CNNM1 (35), FILIP1L (36)], tumor suppressors [VWA5A (37), SYNPO2 (38), ALDH1A2 (39)], and those found overexpressed in certain tumors with unknown function [MPV17 (40)] (**Figures 6D–F**). Tumors treated with tumor-specific IgE-sensitized MCs compared to controls also had several downregulated signaling intermediates including those shown to promote tumor growth and progression in tumor microenvironments [PRSS2 (41), PRSS1 (42), REG4 (43), PRKAR2B (44), NDUFA4L2 (45), LRFN5 (46), and ANGPTL4 (47)]. These signaling intermediates and pathways provide a unique overview of the mechanisms of MC-induced killing of tumor cells and work is underway to further validate and delineate the anti-tumor mechanisms involved.

### Transduction of ADMC and Adipose Stem Cells With GFP

**Figure 7:**
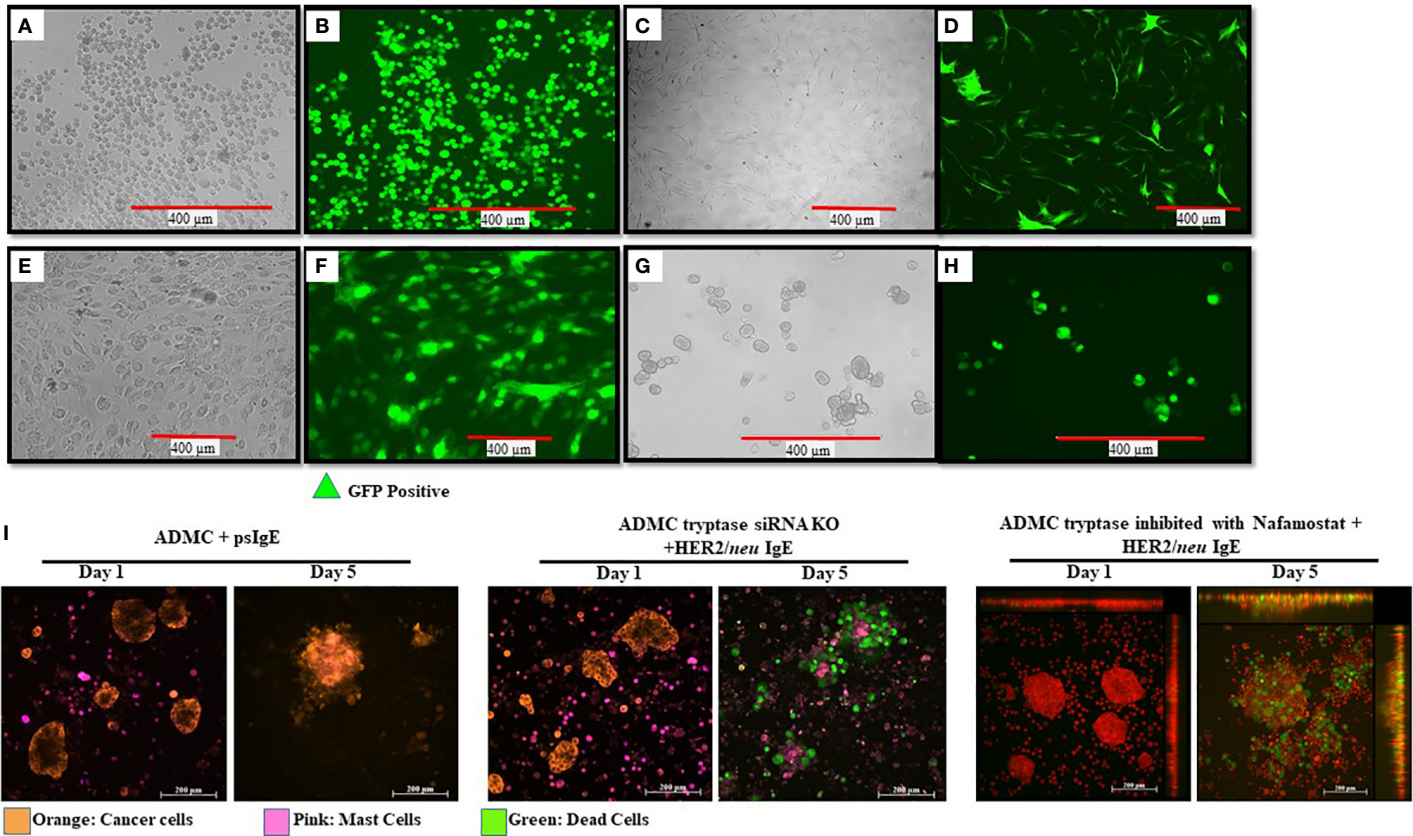
Manipulation of human MCs and adipose stem cells as a platform for increasing safety and cytotoxicity. ADMC (**A, B**; 5x10^6^) or adipose stem cells (**C, D**; 3x10^6^) 3 days after infection by 20 μl of GFP-carrying lentiviral particles (4x10^8^ Infection Unit). Mag. 20x. Adipose-derived stem cells were transduced with GFP, cryopreserved and reconstituted in stem cell medium **(E, F)** and GFP fluorescence. **(G, H)** Culturing in ADMC medium for 4 months showing ADMC with GFP Mag. 20x. **(I)** MCs (2x10^5^) were treated with silencer select siRNA targeting the *TPSAB1* gene, sensitized with psIgE or HER2/*neu* IgE followed by staining with CellTracker™ Deep Red. After washing, MCs were added to the pre-stained, adherent BT474 (1x10^5^ -8x10^5^) along with caspase 3/7 and the time lapse video was taken over the indicated times. Image colors were adjusted using AxioVision software where, blue is changed to orange (BT474) and red is changed to pink (MCs). Results are representative of three different donors.

Little is known about what MC mediators are responsible for the apoptosis of cancer cells upon FcεRI activation and contribute to anti-tumor activity. While we have demonstrated that TNF-α is involved in *in vitro* killing using anti-TNF-α Abs (10), this method is not optimal for *in vivo* assessment. To begin to address the question, we sought to inhibit/knockout selected MC-specific mediators to assess which mediators are anti-tumorigenic. With this information, it might then be possible to polarize and engineer the MCs to remove potential toxic mediators, while retaining (or supplementing) anti-tumor mediators. Our first step was to determine if primary human ADMC could be transduced. As seen in **Figure 7**, it is shown for the first time that primary ADMC (**Figures 7A, B**) and adipose-derived stem cells (which give rise to ADMC; **Figures 7C, D**) maintain viability after being transduced with GFP (under the control of a CMV promoter) *via* a lentivirus system. The transduced ADMC did not lose phenotypic characteristics or FcϵRI functional responses over four months (data not shown). Importantly, GFP-transduced adipose stem cells that had been cryopreserved in liquid nitrogen and reconstituted retained GFP fluorescence (**Figures 7E, F**). These same adipose stem cells differentiated into ADMC retained GFP up to four months (**Figures 7G, H**). The observation that adipose stem cells can be cryopreserved without losing viability has important implications for therapeutic applications.

Experiments were performed to assess select inhibitor/knocking down of MC-specific mediators to determine what human MC mediators exert pro- or anti-tumorigenic effects. Tryptase is a MC-specific mediator that has been reported to have both pro- and anti-tumor effects (48–52). Incubation of MCs with anti-tryptase siRNA was optimal at 20 picomole (**Supplementary Figure 4**). As shown in **Figure 7I** HER2/*neu* IgE-sensitized MCs with tryptase inhibited >95% still exhibited significant apoptosis of BT474-1261 cells. In separate experiments, MCs treated with the tryptase specific inhibitor Nafamostate did not affect their ability to induce apoptosis and also suggested a non-critical role for tryptase in inducing IgE-dependent apoptosis of cancer cells (**Figure 7I**). These studies suggest MC-specific tryptase is not directly involved in tumor cell apoptosis and provide conditions needed so that the role of other MC mediators can be assessed for anti-tumor activity.

## Discussion

The number and diversity of autologous cells being explored for their ability to confer anti-tumor properties continues to increase. The most widely recognized and successful strategy of adoptive cellular transfer (ACT) is the use of autologous, peripheral T cells engineered *ex vivo* to express a transmembrane chimeric antigen receptor composed of an extracellular, antigen-specific single-chain antibody and an intracellular T cell signaling domain (CAR T) (53, 54). In addition to other T-cell bases strategies, the use of non-T immune cells are also being investigated for anti-tumor activity. For example, dendritic cells loaded *in vitro* with specific tumor-associated antigens to generate an immune response for cancer-cell elimination has led to clinical trials testing safety and efficacy (55). Natural killer cells that target cancer stem-like cells can skew the immune response toward anti-tumor activity and are an alternative cell type for CAR therapy (56). Peripheral blood eosinophils and neutrophils have demonstrated anti-tumorigenic activity (57, 58). One of the most promising cell types being explored for ACT are, like MCs, resident in tissue. Macrophages polarized using CAR T cell strategies to redirect their phenotype to M1 with phagocytic functions target cancer-specific biomarkers and induce an adaptive immune response against tumor cells (59). In short, most cells being investigated as new platforms for cancer immunotherapy exert both pro- and anti-tumor effects. Therefore, the challenge moving forward is to identify and utilize these cells and take advantage of current technologies that allow for the removal of pro-tumor activity and/or enhance their anti-tumor functions. Here we present evidence that this can be accomplished with *ex vivo* derived MCs.

Our studies demonstrate that human ADMC have anti-tumor properties *in vitro* and *in vivo* and without overt signs of toxicity. These effects are observed even without the need for the anti-cancer adjuvant GM-CSF as human GM-CSF does not bind cognate mouse receptors (60). The studies utilized a single MC injection and the tumor volumes appeared to fluctuate in the HER2/*neu* IgE-sensitized ADMC animals which we hypothesize could be due in part to the well-documented capability of MCs to re-activate following FcεRI activation to levels similar to what is seen in the initial activation (61). Stem cell factor, the ligand for MCs expressing c-kit, is the principal growth signal for MCs. While MCs derived *ex vivo* have a finite lifespan without SCF, they are also capable of autocrine SCF secretion and activation of KIT (62, 63) which may account for the prolonged anti-tumor effect observed with only a single injection. Of course, more frequent injections of ADMC may be needed for this strategy to increase the anti-tumor effects. We are currently investigating the optimal parameters needed to increase efficacy in these *in vivo* models.

One of the most controversial areas of research in MC-tumor biology is the role MCs have in tumor pathogenesis. While MCs may be able to shrink tumors with “natural” anti-tumor mediators (as demonstrated above), our strategy is to be able to increase efficacy by introducing other proven anti-tumor mediators (or decrease potential toxicity). While we reported that TNF-α was involved in *in vitro* killing of tumor cells using anti-TNF-α Abs (10) it is simply not known what the role other mediators elicited from MCs have in apoptosis induction. Knocking out or knocking down histamine would be an obvious choice for selective removal and alleviate the widespread concerns using cytotoxic MCs for cancer therapy. However, an anti-tumor role for histamine has been suggested (8, 64). If “left in” the MCs, a histamine-mediated reaction is immediate and could be dealt with using anti-histamines as is widely prescribed. If “taken out” of MCs, then the anti-tumor effects may be compromised.

We propose a strategy utilizing genetic knockdown of mediators released upon FcεRI-specific activation followed by testing the resulting MC for their ability to induce apoptosis compared to non-manipulated MCs. Primary human MCs are difficult to genetically manipulate with low yields and poor transfection efficiencies. Imaging blood-derived MC degranulation using single cells subjected to shRNA knockdown and high-resolution confocal microscopy was recently described (65) but this procedure results in very low transfection efficiency using ADMC and skin-derived MCs and thus not suitable for numbers needed for therapeutic applications. Our strategy was thus to first determine which MC mediators elicited following tumor cell-induced, FcεRI crosslinking are active in apoptosis. Then rational decisions can be made for more focused efforts at genetic engineering of *ex vivo* MCs for polarization into less toxic, and/or more potent, cytotoxic cells using the lentivirus system described above which is resistant to mobilization (safer for clinical trials), resistant to epigenetic silencing of non-integrating lentiviral vectors, and is a better strategy for delivery of toxic genes (66). Tryptase was initially selected given its MC specificity, availability of inhibitors, and radical conflicting data as to its role in cancer pathogenesis (49, 50, 52). We show tryptase-deficient MCs still exert potent anti-tumor cell apoptosis suggesting this protease does not have a direct effect on tumor cells. Determination of the conditions needed for manipulating primary MC genes, selection of targets, along with the observation that adipose stem cells can be cryopreserved without losing viability (**Figure 7**), has important implications for therapeutic strategies. This opens the possibility to add a wide range of anti-tumor mediators in addition to the “naturally occurring” anti-tumor mediators in MCs (already demonstrated to shrink tumors and extend lifespan) to develop a “super killing” cell for cancer (or other malignancies in which there are IgE targets) immunotherapy.

These are the first reports of primary human FcεRI-activated MC forming TnT with tumor cells in parallel with apoptosis. The TnT have been described in multiple cell types *in vitro* and *in vivo* as a form of cell-to-cell communication in a variety of disease mechanisms, including cancer (67). These thin plasma membrane structures connecting cells can transfer a wide array of molecules, including organelles and small molecules. Previous studies using the MC line LAD reported the emergence of TnT when co-cultured with glioblastoma cells (68). These transformed, immortal cells were not challenged through FcεRI as performed with primary human MCs described here. The significance of the TnT formation between MCs and cancer cells *in vitro* is still unclear. Currently we are investigating the possibility that mediators and/or organelles are transferred between cells, if MCs form TnT *in vitro* using cancer cell spheroids models, and *in vivo* using patient-derived xenograft models in immunocompromised mice to determine any correlations between TnT formation and their ability to induce tumor shrinkage or alter their function and susceptibility to therapeutics.

The induction of MC-induced apoptosis of tumor cells appears to be bi-directional with apoptotic bodies forming at the junctions of the MC and cancer cells (**Figure 3**). Apoptotic bodies are a type of small, membrane-bound extracellular vesicle that range from 50 to 5,000 nm in diameter that are produced from cancer cells undergoing programmed cell death (69). These bodies form during plasma membrane blebbing following an apoptotic signal and can release them as extracellular vesicles that can carry nuclear fragments and cellular organelles such as mitochondria and endoplasmic reticulum for further immune responses (70). In addition, MCs clearly enter and migrate through tumor cell bundles and become activated (**Figure 3** and **Supplementary Video 1**), suggesting they may have the ability to enter tumors to release their mediators. This is intriguing as most ACT strategies are not as effective on solid tumors as they are not able to penetrate the outer cell layer.

TNF-α was investigated early on as a potential anti-tumor therapy based on its ability to necrotize mouse tumors (71). Toxicity associated with systemic TNF administration or release beyond the tumor milieu remains a problem which could be addressed if specific and directed release to cancer cells could be attained (71). Clinical trials utilizing systemic TNF-α administration have resulted in unacceptable level of toxicities, which blocked its development (72). In contrast, localized administration of TNF-α in the form of isolated limb perfusion have yielded excellent results in soft tissue sarcomas (73). Our results suggest TNF-α plays a significant role in the MC-induced apoptosis of cancer cells and the strategy using cytotoxic MCs with pre-made TNF-α within the granules is exactly what is needed for utilizing TNF-α as an anti-cancer agent; targeted, localized, and controlled release only upon tumor cell engagement. In fact-we provide evidence that this pre-formed mediator is released within the tumor cells further enhancing its effect (**Figure 3** and data not shown) and possibly alleviating the toxicity encountered with systemic release. MCs could provide the exact solution to TNF-α systemic toxicity by selectively releasing it, controlled only by tumor cell engagement, in and around the tumor cells.

The gene analysis of mRNA within tumor cells affected by cytotoxic MCs revealed a plethora of information suggesting a completely new and diverse mechanism of tumor cell killing. In general, there was a significant upregulation of genes previously demonstrated to have anti-tumor activity and downregulation of many having pro-tumor involvement. For example, TNF-related apoptosis-inducing ligand (TRAIL) is a potent stimulator of apoptosis, especially on tumor cells, making them excellent therapeutic targets for cancer (74). Cytotoxic ADMC upregulated several TRAIL receptors (e.g. TNFRSF10) on tumor cells suggesting MCs could provide external triggers for apoptosis. The NADH dehydrogenase 1 alpha subcomplex, 4-like 2 (NDUFA4L2) is important in progression of multiple cancers and educed expression through lentivirus knockdown led to a significant enhancement of tumor cell apoptosis *in vitro* and *in vivo (* 45). We show NDUFA4L2 was significantly downregulated (2.5 fold) in tumors challenged with MCs. The serpin family A member 1 (SERPINA1) gene is upregulated and is a marker of poor prognosis for several cancers (75) which was also significantly downregulated in tumor cells following ADMC challenge (1.4 fold). The metallophosphoesterase domain containing 2 protein (MPPED2) was shown to be a potent tumor suppressor involved in the downregulation of breast carcinogenesis (33) and ADMC significantly upregulated (2.0 fold) it in tumor cells. These data suggest MCs induce a unique and potent anti-tumor response through a diversity of anti-tumor pathways leading to apoptosis. We are currently following up on many of these targets affected by cytotoxic MCs to understand their role in tumor cell apoptosis and function.

Similar to those cell types mentioned above being investigated for ACT, MCs have pro- and anti-tumor mediators which has led to speculation and conjecture as to their role in cancer pathogenesis. Depleting or stabilizing MCs has been hypothesized to enhance anti-cancer mechanisms (76). As with MCs, it was hypothesized tumor macrophage depletion and/or inhibition was also a plausible strategy for anti-cancer therapeutics (77). Now, macrophages are at the forefront of translational ACT research and are poised to become another cell in the arsenal of cancer cell immunotherapies (78). Of course, there are a myriad of logical concerns and arguments as to why ACT with MCs is a challenging strategy. Yet, similar arguments against CAR T were also raised even up until clinical trials ensued which resulted in the now manageable cytokine storm driven by IL-6 (79).

Personalized medicine using a cancer patient’s own immune system to direct anti-tumor effects is an exciting and growing area of research. MCs possess anti-tumor mediators, can be obtained from autologous sources, and cryopreserved. We exploited the availability of tumor-targeting IgE’s that arm the MCs for highly efficient and controlled release upon tumor cell engagement that results in tumor shrinkage and lifespan extension without the systemic release of Type I hypersensitivity mediators. The MCs induce a unique anti-tumor mechanism with upregulation of several anti-tumor genes and downregulation of several pro-tumor genes. Additionally, we have revealed a strategy to identify which MC mediators are responsible for tumor regression and those that are not so that gene transfer methods can polarize MCs to be more potent anti-cancer cells with less potential toxicity potential. The significant anti-tumor activity by MCs revealed a heretofore undescribed effect on increasing tumor cell suppressor genes and decreasing pro-tumor genes. This autologous MC platform has thus the potential to be used against many cancers for which tumor IgE’s are available or could be manufactured.

## Data Availability Statement

The data presented in the study are deposited in the European Nucleotide Archive (ENA) repository, accession number PRJEB51596.

## Ethics Statement

The animal study was reviewed and approved by UNCG IRB.

## Author Contributions

Conceptualization: CK. Methodology: MF, EA, PD, MM, KD, YY, SL, AB, TK, and CK. Investigation: MF, EA, MM, KD, YY, SL, AB, TK, DM, and CK. Writing-original draft: MF and CK. Writing-review and editing: MF, EA, MM, KD, AD, YY, SL, AB, TK, DM, and CK. Funding acquisition: CK and TK. Resources: DM, AB, TK, and CK. Supervision: DM, AB, TK, and CK. All authors contributed to the article and approved the submitted version.

## Funding

CK was funded by NIH/NCI grant number 1R15CA246430, Specialized Center of Research grant and Pilot grant from UNC-Chapel Hill, Lineberger Cancer Center. DM and YY are supported by the Division of Intramural Research, NIH/NIAID. AB and the Mouse Phase 1 Unit are supported by the University Cancer Research Fund (UCRF).

## Conflict of Interest

The authors declare that the research was conducted in the absence of any commercial or financial relationships that could be construed as a potential conflict of interest.

## Publisher’s Note

All claims expressed in this article are solely those of the authors and do not necessarily represent those of their affiliated organizations, or those of the publisher, the editors and the reviewers. Any product that may be evaluated in this article, or claim that may be made by its manufacturer, is not guaranteed or endorsed by the publisher.

## Supplementary Material

Supplementary Video 1 Adipose-derived MCs bind to and induce apoptosis of tumor cells. Experiment is described in Figure 3.

Supplementary Video 2 Peripheral blood-derived MCs bind to and induce apoptosis of tumor cells. Experiment is described in Figure 3.

Supplementary Video 3
*In vivo* imaging of HER2/*neu* IgE-sensitized MCs binding to and shrinking HER2/*neu* tumors.

Supplementary Video 4 Representative video of mice injected i.v. with HER2/*neu* IgE-sensitized MCs (1 x 10^6^) into HER2/*neu* tumor engrafted mice. Images were obtained at approximately 20 minutes after tail vein injection and demonstrates no anaphylaxis.

## Abbreviations

MCs, Mast cells; BC, Breast cancer; ADMC, Adipose-Derived mast cells; BDMC, Peripheral blood derived mast cells; HER2/*neu*, Human epidermal growth factor receptor 2; FcεRI, High affinity receptor for IgE; IgE, Immunoglobin E; TGF, Transforming growth factor; TNF-α, Tumor necrosis factor alpha; GM-CSF, Granulocyte-macrophage colony-stimulating factor; ACT, Adoptive cell transfer; CAR T, Chimeric antigen receptor t cells.

## References

[1] Varricchi G Galdiero MR Loffredo S Marone G Iannone R Granata F. Are Mast Cells MASTers in Cancer? Front Immunol (2017) 8:424. doi: 10.3389/fimmu.2017.00424 28446910 PMC5388770

[2] Derakhshani A Vahidian F Alihasanzadeh M Mokhtarzadeh A Lotfi Nezhad P Baradaran B. Mast Cells: A Double-Edged Sword in Cancer. Immunol Lett (2019) 209:28–35. doi: 10.1016/j.imlet.2019.03.011 30905824

[3] Wenzel S Holgate ST. The Mouse Trap: It Still Yields Few Answers in Asthma. Am J Respir Crit Care Med (2006) 174(11):1173. doi: 10.1164/rccm.2609002 17110654

[4] Rodewald HR Feyerabend TB. Widespread Immunological Functions of Mast Cells: Fact or Fiction? Immunity (2012) 37(1):13–24. doi: 10.1016/j.immuni.2012.07.007 22840840

[5] Mestas J Hughes CC. Of Mice and Not Men: Differences Between Mouse and Human Immunology. J Immunol (2004) 172(5):2731–8. doi: 10.4049/jimmunol.172.5.2731 14978070

[6] Bruhns P. Properties of Mouse and Human IgG Receptors and Their Contribution to Disease Models. Blood (2012) 119(24):5640–9. doi: 10.1182/blood-2012-01-380121 22535666

[7] Massari NA Nicoud MB Medina VA. Histamine Receptors and Cancer Pharmacology: An Update. Br J Pharmacol (2020) 177(3):516–38. doi: 10.1111/bph.14535 PMC7012953 30414378

[8] Nicoud MB Sterle HA Massari NA Taquez Delgado MA Formoso K Herrero Ducloux MV. Study of the Antitumour Effects and the Modulation of Immune Response by Histamine in Breast Cancer. Br J Cancer (2020) 122(3):348–60. doi: 10.1038/s41416-019-0636-x PMC7000401 31748740

[9] Gordon JR Galli SJ. Mast Cells as a Source of Both Preformed and Immunologically Inducible TNF-‡/Cachectin. Nature (1990) 346:274–6. doi: 10.1038/346274a0 2374592

[10] Plotkin JD Elias MG Fereydouni M Daniels-Wells TR Dellinger AL Penichet ML. Human Mast Cells From Adipose Tissue Target and Induce Apoptosis of Breast Cancer Cells. Front Immunol (2019) 10:138. doi: 10.3389/fimmu.2019.00138 30833944 PMC6387946

[11] Josephs SF Ichim TE Prince SM Kesari S Marincola FM Escobedo AR. Unleashing Endogenous TNF-Alpha as a Cancer Immunotherapeutic. J Trans Med (2018) 16(1):242. doi: 10.1186/s12967-018-1611-7 PMC6119315 30170620

[12] Yan WL Shen KY Tien CY Chen YA Liu SJ. Recent Progress in GM-CSF-Based Cancer Immunotherapy. Immunotherapy (2017) 9(4):347–60. doi: 10.2217/imt-2016-0141 28303764

[13] Zahavi D Weiner L. Monoclonal Antibodies in Cancer Therapy. Antibodies (2020) 9(3):34. doi: 10.3390/antib9030034 PMC7551545 32698317

[14] Leoh LS Daniels-Wells TR Penichet ML. IgE Immunotherapy Against Cancer. Curr Top Microbiol Immunol (2015) 388:109–49. doi: 10.1007/978-3-319-13725-4_6 PMC4452505 25553797

[15] Chauhan J McCraw AJ Nakamura M Osborn G Sow HS Cox VF. IgE Antibodies Against Cancer: Efficacy and Safety. Antibodies (2020) 9(4):55. doi: 10.3390/antib9040055 PMC7709114 33081206

[16] Turner H Kinet JP. Signalling Through the High-Affinity IgE Receptor Fc epsilonRI. Nature (1999) 402(6760 Suppl):B24–30. doi: 10.1038/35037021 10586892

[17] Varricchi G Galdiero MR Loffredo S Marone G Iannone R Marone G. Are Mast Cells MASTers in Cancer? Front Immunol (2017) 8:424. doi: 10.3389/fimmu.2017.00424 28446910 PMC5388770

[18] Teo PZ Utz PJ Mollick JA. Using the Allergic Immune System to Target Cancer: Activity of IgE Antibodies Specific for Human CD20 and MUC1. Cancer Immunol Immunother (2012) 61(12):2295–309. doi: 10.1007/s00262-012-1299-0 PMC3833261 22692757

[19] Hudis CA. Trastuzumab–Mechanism of Action and Use in Clinical Practice. N Engl J Med (2007) 357(1):39–51. doi: 10.1056/NEJMra043186 17611206

[20] Yin Y Bai Y Olivera A Desai A Metcalfe DD. An Optimized Protocol for the Generation and Functional Analysis of Human Mast Cells From CD34(+) Enriched Cell Populations. J Immunol Methods (2017) 448:105–11. doi: 10.1016/j.jim.2017.06.003 PMC5654741 28629733

[21] Norton SK Dellinger A Zhou Z Lenk R Macfarland D Vonakis B. A New Class of Human Mast Cell and Peripheral Blood Basophil Stabilizers That Differentially Control Allergic Mediator Release. Clin Transl Sci (2010) 3(4):158–69. doi: 10.1111/j.1752-8062.2010.00212.x PMC5350695 20718816

[22] Dellinger A Olson J Link K Vance S Sandros MG Yang J. Functionalization of Gadolinium Metallofullerenes for Detecting Atherosclerotic Plaque Lesions by Cardiovascular Magnetic Resonance. J Cardiovasc Magnetic Resonance: Off J Soc Cardiovasc Magnetic Resonance (2013) 15:7. doi: 10.1186/1532-429X-15-7 PMC3562260 23324435

[23] Irani AM Bradford TR Kepley CL Schechter NM Schwartz LB. Detection of MCT and MCTC Types of Human Mast Cells by Immunohistochemistry Using New Monoclonal Anti-Tryptase and Anti-Chymase Antibodies. J Histochem Cytochem (1989) 37(10):1509–15. doi: 10.1177/37.10.2674273 2674273

[24] Schaer DA Hirschhorn-Cymerman D Wolchok JD. Targeting Tumor-Necrosis Factor Receptor Pathways for Tumor Immunotherapy. J Immunother Cancer (2014) 2(7):2051–1426. doi: 10.1186/2051-1426-2-7 PMC4030310 24855562

[25] Zhang YQ Zhang JJ Song HJ Li DW. Overexpression of CST4 Promotes Gastric Cancer Aggressiveness by Activating the ELFN2 Signaling Pathway. Am J Cancer Res (2017) 7(11):2290–304. PMC5714756 29218251

[26] Bai Y Sun Y Peng J Liao H Gao H Guo Y. Overexpression of Secretagogin Inhibits Cell Apoptosis and Induces Chemoresistance in Small Cell Lung Cancer Under the Regulation of miR-494. Oncotarget (2014) 5(17):7760–75. doi: 10.18632/oncotarget.2305 PMC4202159 25226615

[27] Ostrovskaya A Hick C Hutchinson DS Stringer BW Wookey PJ Wootten D. Expression and Activity of the Calcitonin Receptor Family in a Sample of Primary Human High-Grade Gliomas. BMC Cancer (2019) 19(1):157. doi: 10.1186/s12885-019-5369-y 30777055 PMC6379965

[28] Wu DC Zhang MF Su SG Fang HY Wang XH He D. HEY2, a Target of miR-137, Indicates Poor Outcomes and Promotes Cell Proliferation and Migration in Hepatocellular Carcinoma. Oncotarget (2016) 7(25):38052–63. doi: 10.18632/oncotarget.9343 PMC5122371 27191260

[29] Liu Z Sanders AJ Liang G Song E Jiang WG Gong C. Hey Factors at the Crossroad of Tumorigenesis and Clinical Therapeutic Modulation of Hey for Anticancer Treatment. Mol Cancer Ther (2017) 16(5):775–86. doi: 10.1158/1535-7163.MCT-16-0576 28468863

[30] Kong Y Ou X Li X Zeng Y Gao G Lyu N. LGR6 Promotes Tumor Proliferation and Metastasis Through Wnt/β-Catenin Signaling in Triple-Negative Breast Cancer. Mol Ther Oncolytics (2020) 18:351–9. doi: 10.1016/j.omto.2020.06.020 PMC7403884 32775619

[31] Mamoor S. MATK Is Differentially Expressed in the Tumors of Breast Cancer Patients Treated With Trastuzumab. Charlottesville, Virginia: OSF (Center for Open Science) (2020). doi: 10.31219/osf.io/sj2fb

[32] Maertens O Cichowski K. An Expanding Role for RAS GTPase Activating Proteins (RAS GAPs) in Cancer. Adv Biol Regul (2014) 55:1–14. doi: 10.1016/j.jbior.2014.04.002 24814062

[33] Pellecchia S Sepe R Federico A Cuomo M Credendino SC Pisapia P. The Metallophosphoesterase-Domain-Containing Protein 2 (MPPED2) Gene Acts as Tumor Suppressor in Breast Cancer. Cancers (2019) 11(6):797. doi: 10.3390/cancers11060797 PMC6627064 31181813

[34] Han S Yang Y. Interleukin-32: Frenemy in Cancer? BMB Rep (2019) 52(3):165–74. doi: 10.5483/BMBRep.2019.52.3.019 PMC6476484 30638183

[35] Huang Y Huang H Han Z Li W Mai Z Yuan R. Ginsenoside Rh2 Inhibits Angiogenesis in Prostate Cancer by Targeting Cnnm1. J Nanosci Nanotechnol (2019) 19(4):1942–50. doi: 10.1166/jnn.2019.16404 30486934

[36] Park YL Park SY Lee SH Kim RB Kim JK Rew SY. Filamin A Interacting Protein 1-Like Expression Inhibits Progression in Colorectal Cancer. Oncotarget (2016) 7(44):72229–41. doi: 10.18632/oncotarget.12664 PMC5342157 27750216

[37] Martin ES Cesari R Pentimalli F Yoder K Fishel R Himelstein AL. The BCSC-1 Locus at Chromosome 11q23-Q24 is a Candidate Tumor Suppressor Gene. Proc Natl Acad Sci USA (2003) 100(20):11517–22. doi: 10.1073/pnas.1934602100 PMC208790 14504409

[38] Liu J Ye L Li Q Wu X Wang B Ouyang Y. Synaptopodin-2 Suppresses Metastasis of Triple-Negative Breast Cancer *via* Inhibition of YAP/TAZ Activity. J Pathol (2018) 244(1):71–83. doi: 10.1002/path.4995 28991374

[39] Choi JA Kwon H Cho H Chung JY Hewitt SM Kim JH. ALDH1A2 Is a Candidate Tumor Suppressor Gene in Ovarian Cancer. Cancers (2019) 11(10):1553. doi: 10.3390/cancers11101553 PMC6826427 31615043

[40] Canonne M Wanet A Nguyen TTA Khelfi A Ayama-Canden S Van Steenbrugge M. MPV17 Does Not Control Cancer Cell Proliferation. PloS One (2020) 15(3):e0229834–e. doi: 10.1371/journal.pone.0229834 PMC7064194 32155188

[41] Sui L Wang S Ganguly D Raye TE Askeland C Børretzen A. PRSS2 Promotes Tumor Growth and Progression by Repressing Tsp-1 in the Tumor Microenvironment *via* Binding to LRP1. bioRxiv (2021). doi: 10.1101/2021.03.23.436667.

[42] Liu Q Guo L Zhang S Wang J Lin X Gao F. PRSS1 Mutation: A Possible Pathomechanism of Pancreatic Carcinogenesis and Pancreatic Cancer. Mol Med (2019) 25(1):019–0111. doi: 10.1186/s10020-019-0111-4 PMC6744682 31521106

[43] Zhang J Zhu Z Miao Z Huang X Sun Z Xu H. The Clinical Significance and Mechanisms of REG4 in Human Cancers. Front Oncol (2021) 10:559230. doi: 10.3389/fonc.2020.559230 PMC7819868 33489872

[44] Sha J Han Q Chi C Zhu Y Pan J Dong B. PRKAR2B Promotes Prostate Cancer Metastasis by Activating Wnt/β-Catenin and Inducing Epithelial-Mesenchymal Transition. J Cell Biochem (2018) 119(9):7319–27. doi: 10.1002/jcb.27030 29761841

[45] Chen Z Wei X Wang X Zheng X Chang B Shen L. NDUFA4L2 Promotes Glioblastoma Progression, Is Associated With Poor Survival, and can be Effectively Targeted by Apatinib. Cell Death Dis (2021) 12(4):377. doi: 10.1038/s41419-021-03646-3 33828084 PMC8027655

[46] Sakthikumar S Roy A Haseeb L Pettersson ME Sundström E Marinescu VD. Whole-Genome Sequencing of Glioblastoma Reveals Enrichment of Non-Coding Constraint Mutations in Known and Novel Genes. Genome Biol (2020) 21(1):127. doi: 10.1186/s13059-020-02035-x 32513296 PMC7281935

[47] Tan MJ Teo Z Sng MK Zhu P Tan NS. Emerging Roles of Angiopoietin-Like 4 in Human Cancer. Mol Cancer Res (2012) 10(6):677. doi: 10.1158/1541-7786.MCR-11-0519 22661548

[48] Schwartz LB. Tryptase: A Clinical Indicator of Mast Cell-Dependent Events. Allergy Proc (1994) 15:119–23. doi: 10.2500/108854194778702946 7926709

[49] Ammendola M Leporini C Marech I Gadaleta CD Scognamillo G Sacco R. Targeting Mast Cells Tryptase in Tumor Microenvironment: A Potential Antiangiogenetic Strategy. BioMed Res Int (2014) 2014:154702. doi: 10.1155/2014/154702 25295247 PMC4177740

[50] Ranieri G Ammendola M Patruno R Celano G Zito FA Montemurro S. Tryptase-Positive Mast Cells Correlate With Angiogenesis in Early Breast Cancer Patients. Int J Oncol (2009) 35(1):115–20. doi: 10.3892/ijo_00000319 19513558

[51] Blair RJ Meng H Marchese MJ Ren S Schwartz LB Tonnesen MG. Human Mast Cells Stimulate Vascular Tube Formation. Tryptase Is a Novel, Potent Angiogenic Factor. J Clin Invest (1997) 99(11):2691–700. doi: 10.1172/JCI119458 PMC508115 9169499

[52] Rabelo Melo F Santosh Martin S Sommerhoff CP Pejler G. Exosome-Mediated Uptake of Mast Cell Tryptase Into the Nucleus of Melanoma Cells: A Novel Axis for Regulating Tumor Cell Proliferation and Gene Expression. Cell Death Dis (2019) 10(9):659. doi: 10.1038/s41419-019-1879-4 31506436 PMC6736983

[53] Mohanty R Chowdhury CR Arega S Sen P Ganguly P Ganguly N. CAR T Cell Therapy: A New Era for Cancer Treatment (Review). Oncol Rep (2019) 42(6):2183–95. doi: 10.3892/or.2019.7335 31578576

[54] June CH O’Connor RS Kawalekar OU Ghassemi S Milone MC. CAR T Cell Immunotherapy for Human Cancer. Science (2018) 359(6382):1361–5. doi: 10.1126/science.aar6711 29567707

[55] Huber A Dammeijer F Aerts J Vroman H. Current State of Dendritic Cell-Based Immunotherapy: Opportunities for *In Vitro* Antigen Loading of Different DC Subsets? Front Immunol (2018) 9:2804. doi: 10.3389/fimmu.2018.02804 30559743 PMC6287551

[56] Shimasaki N Jain A Campana D. NK Cells for Cancer Immunotherapy. Nat Rev Drug Discov (2020) 19(3):200–18. doi: 10.1038/s41573-019-0052-1 31907401

[57] Reichman H Itan M Rozenberg P Yarmolovski T Brazowski E Varol C. Activated Eosinophils Exert Antitumorigenic Activities in Colorectal Cancer. Cancer Immunol Res (2019) 7(3):388–400. doi: 10.1158/2326-6066.CIR-18-0494 30665890

[58] Furumaya C Martinez-Sanz P Bouti P Kuijpers TW Matlung HL. Plasticity in Pro- and Anti-Tumor Activity of Neutrophils: Shifting the Balance. Front Immunol (2020) 11:2100. doi: 10.3389/fimmu.2020.02100 32983165 PMC7492657

[59] Klichinsky M Ruella M Shestova O Lu XM Best A Zeeman M. Human Chimeric Antigen Receptor Macrophages for Cancer Immunotherapy. Nat Biotechnol (2020) 8 (4_supplement):Abstract nr PR07. doi: 10.1158/2326-6074.TUMIMM18-PR07 PMC7883632 32361713

[60] Manz MG. Human-Hemato-Lymphoid-System Mice: Opportunities and Challenges. Immunity (2007) 26(5):537–41. doi: 10.1016/j.immuni.2007.05.001 17521579

[61] Xiang Z Block M Löfman C Nilsson G. IgE-Mediated Mast Cell Degranulation and Recovery Monitored by Time-Lapse Photography. J Allergy Clin Immunol (2001) 108(1):116–21. doi: 10.1067/mai.2001.116124 11447391

[62] Kitaura J Kinoshita T Matsumoto M Chung S Kawakami Y Leitges M. IgE- and IgE+Ag-Mediated Mast Cell Migration in an Autocrine/Paracrine Fashion. Blood (2005) 105(8):3222–9. doi: 10.1182/blood-2004-11-4205 PMC1464406 15637135

[63] Patella V Marinò I Arbustini E Lamparter-Schummert B Verga L Adt M. Stem Cell Factor in Mast Cells and Increased Mast Cell Density in Idiopathic and Ischemic Cardiomyopathy. Circulation (1998) 97(10):971–8. doi: 10.1161/01.CIR.97.10.971 9529265

[64] Yang XD Ai W Asfaha S Bhagat G Friedman RA Jin G. Histamine Deficiency Promotes Inflammation-Associated Carcinogenesis Through Reduced Myeloid Maturation and Accumulation of CD11b+Ly6G+ Immature Myeloid Cells. Nat Med (2011) 17(1):87–95. doi: 10.1038/nm.2278 21170045 PMC3075560

[65] Folkerts J Gaudenzio N Maurer M Hendriks RW Stadhouders R Tam SY. Rapid Identification of Human Mast Cell Degranulation Regulators Using Functional Genomics Coupled to High-Resolution Confocal Microscopy. Nat Protoc (2020) 15(3):1285–310. doi: 10.1038/s41596-019-0288-6 PMC7197894 32060492

[66] Hu P Bi Y Ma H Suwanmanee T Zeithaml B Fry NJ. Superior Lentiviral Vectors Designed for BSL-0 Environment Abolish Vector Mobilization. Gene Ther (2018) 25(7):454–72. doi: 10.1038/s41434-018-0039-2 PMC6478381 30190607

[67] Tiwari V Koganti R Russell G Sharma A Shukla D. Role of Tunneling Nanotubes in Viral Infection, Neurodegenerative Disease, and Cancer. Front Immunol (2021) 12:680891. doi: 10.3389/fimmu.2021.680891 34194434 PMC8236699

[68] Weng Z Zhang B Tsilioni I Theoharides TC. Nanotube Formation: A Rapid Form of “Alarm Signaling”? Clin Ther (2016) 38(5):1066–72. doi: 10.1016/j.clinthera.2016.02.030 27085584

[69] Kakarla R Hur J Kim YJ Kim J Chwae Y-J. Apoptotic Cell-Derived Exosomes: Messages From Dying Cells. Exp Mol Med (2020) 52(1):1–6. doi: 10.1038/s12276-019-0362-8 31915368 PMC7000698

[70] Poon IKH Lucas CD Rossi AG Ravichandran KS. Apoptotic Cell Clearance: Basic Biology and Therapeutic Potential. Nat Rev Immunol (2014) 14(3):166–80. doi: 10.1038/nri3607 PMC4040260 24481336

[71] Wang X Lin Y. Tumor Necrosis Factor and Cancer, Buddies or Foes? Acta Pharmacol Sin (2008) 29(11):1275–88. doi: 10.1111/j.1745-7254.2008.00889.x PMC2631033 18954521

[72] Chen AY Wolchok JD Bass AR. TNF in the Era of Immune Checkpoint Inhibitors: Friend or Foe? Nat Rev Rheumatol (2021) 17(4):213–23. doi: 10.1038/s41584-021-00584-4 PMC8366509 33686279

[73] Grunhagen DJ de Wilt JH ten Hagen TL Eggermont AM. Technology Insight: Utility of TNF-Alpha-Based Isolated Limb Perfusion to Avoid Amputation of Irresectable Tumors of the Extremities. Nat Clin Pract Oncol (2006) 3(2):94–103. doi: 10.1038/ncponc0426 16462850

[74] Vanamee ÉS Faustman DL. On the TRAIL of Better Therapies: Understanding TNFRSF Structure-Function. Cells (2020) 9(3):764. doi: 10.3390/cells9030764 PMC7140660 32245106

[75] Ercetin E Richtmann S Delgado BM Gomez-Mariano G Wrenger S Korenbaum E. Clinical Significance of SERPINA1 Gene and Its Encoded Alpha1-Antitrypsin Protein in NSCLC. Cancers (2019) 11(9):1306. doi: 10.3390/cancers11091306 PMC6770941 31487965

[76] Oldford SA Marshall JS. Mast Cells as Targets for Immunotherapy of Solid Tumors. Mol Immunol (2015) 63(1):113–24. doi: 10.1016/j.molimm.2014.02.020 24698842

[77] Cotechini T Atallah A Grossman A. Tissue-Resident and Recruited Macrophages in Primary Tumor and Metastatic Microenvironments: Potential Targets in Cancer Therapy. Cells (2021) 10(4):960. doi: 10.3390/cells10040960 33924237 PMC8074766

[78] Anderson NR Minutolo NG Gill S Klichinsky M. Macrophage-Based Approaches for Cancer Immunotherapy. Cancer Res (2021) 81(5):1201–8. doi: 10.1158/0008-5472.CAN-20-2990 33203697

[79] Rosenbaum L. Tragedy, Perseverance, and Chance - The Story of CAR-T Therapy. N Engl J Med (2017) 377(14):1313–5. doi: 10.1056/NEJMp1711886 28902570

