# Supplementary material for: Human Tumor Targeted Cytotoxic Mast Cells for Cancer Immunotherapy
Source: Front Oncol. 2022 Apr 22;12:871390. doi: 10.3389/fonc.2022.871390 (PMC9097604; doi:10.3389/fonc.2022.871390)
Supplement: Supplementary file 1 [file Presentation_1.pptx]

## Slide 1
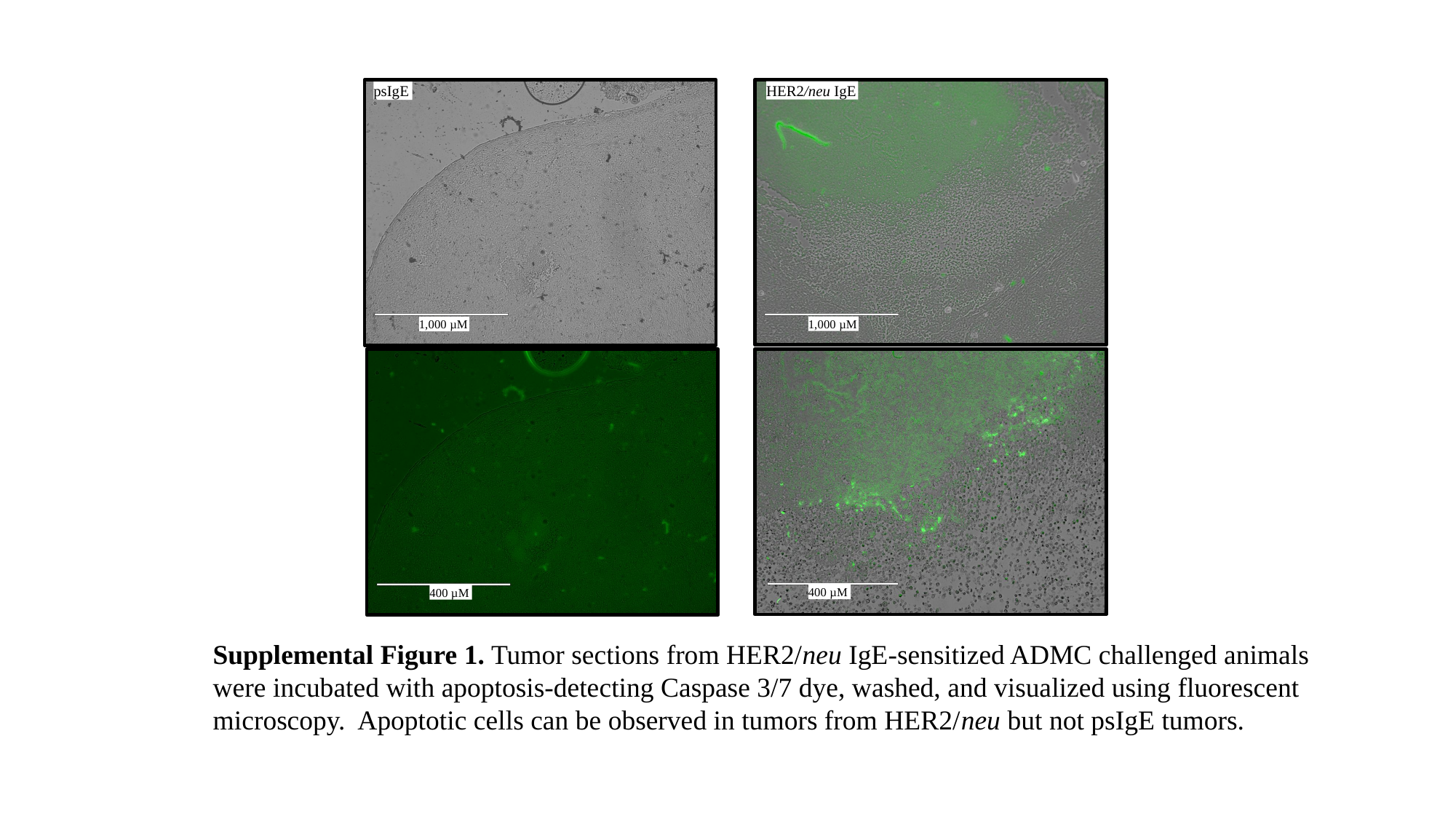

HER2/neu IgE
psIgE
1,000 µM
1,000 µM
400 µM
400 µM
Supplemental Figure 1. Tumor sections from HER2/neu IgE-sensitized ADMC challenged animals were incubated with apoptosis-detecting Caspase 3/7 dye, washed, and visualized using fluorescent microscopy. Apoptotic cells can be observed in tumors from HER2/neu but not psIgE tumors.

## Slide 2
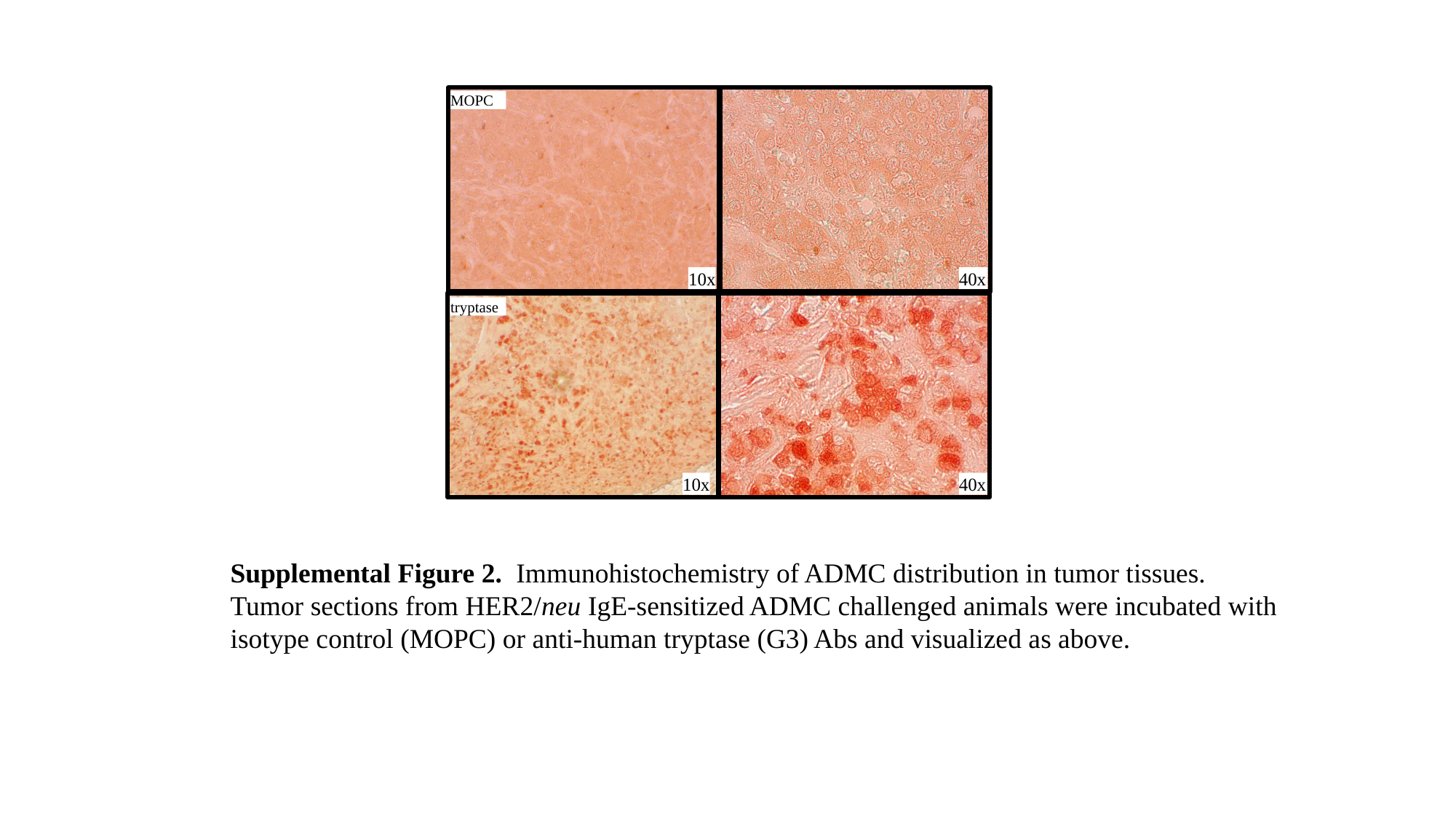

MOPC
10x
40x
tryptase
10x
40x
Supplemental Figure 2. Immunohistochemistry of ADMC distribution in tumor tissues. Tumor sections from HER2/neu IgE-sensitized ADMC challenged animals were incubated with isotype control (MOPC) or anti-human tryptase (G3) Abs and visualized as above.

## Slide 3
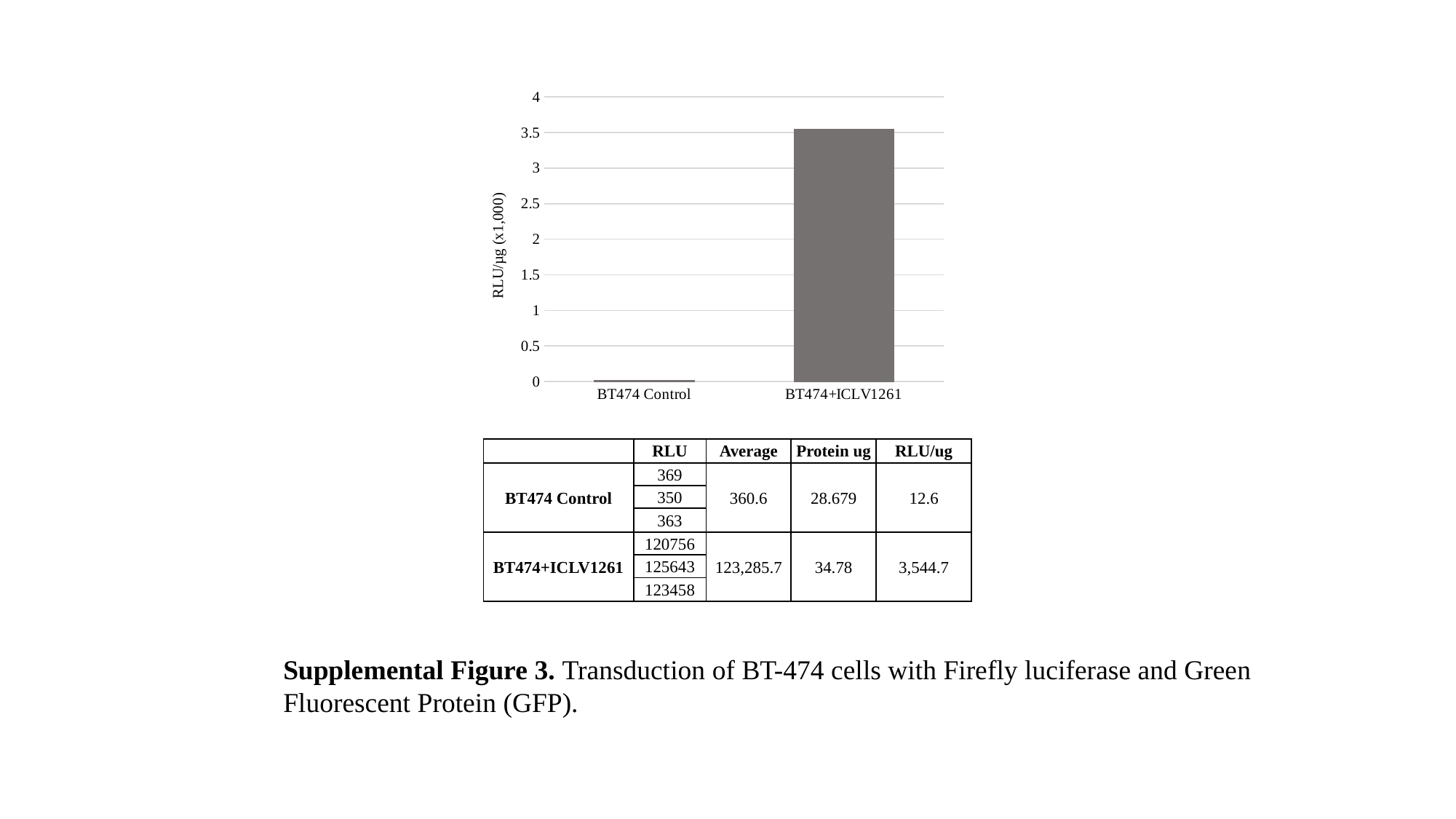

### Chart
| Category | |
|---|---|
| BT474 Control | 0.012575984750746774 |
| BT474+ICLV1261 | 3.544728771324516 || | RLU | Average | Protein ug | RLU/ug |
| --- | --- | --- | --- | --- |
| BT474 Control | 369 | 360.6 | 28.679 | 12.6 |
| | 350 | | | |
| | 363 | | | |
| BT474+ICLV1261 | 120756 | 123,285.7 | 34.78 | 3,544.7 |
| | 125643 | | | |
| | 123458 | | | |
Supplemental Figure 3. Transduction of BT-474 cells with Firefly luciferase and Green Fluorescent Protein (GFP).

## Slide 4
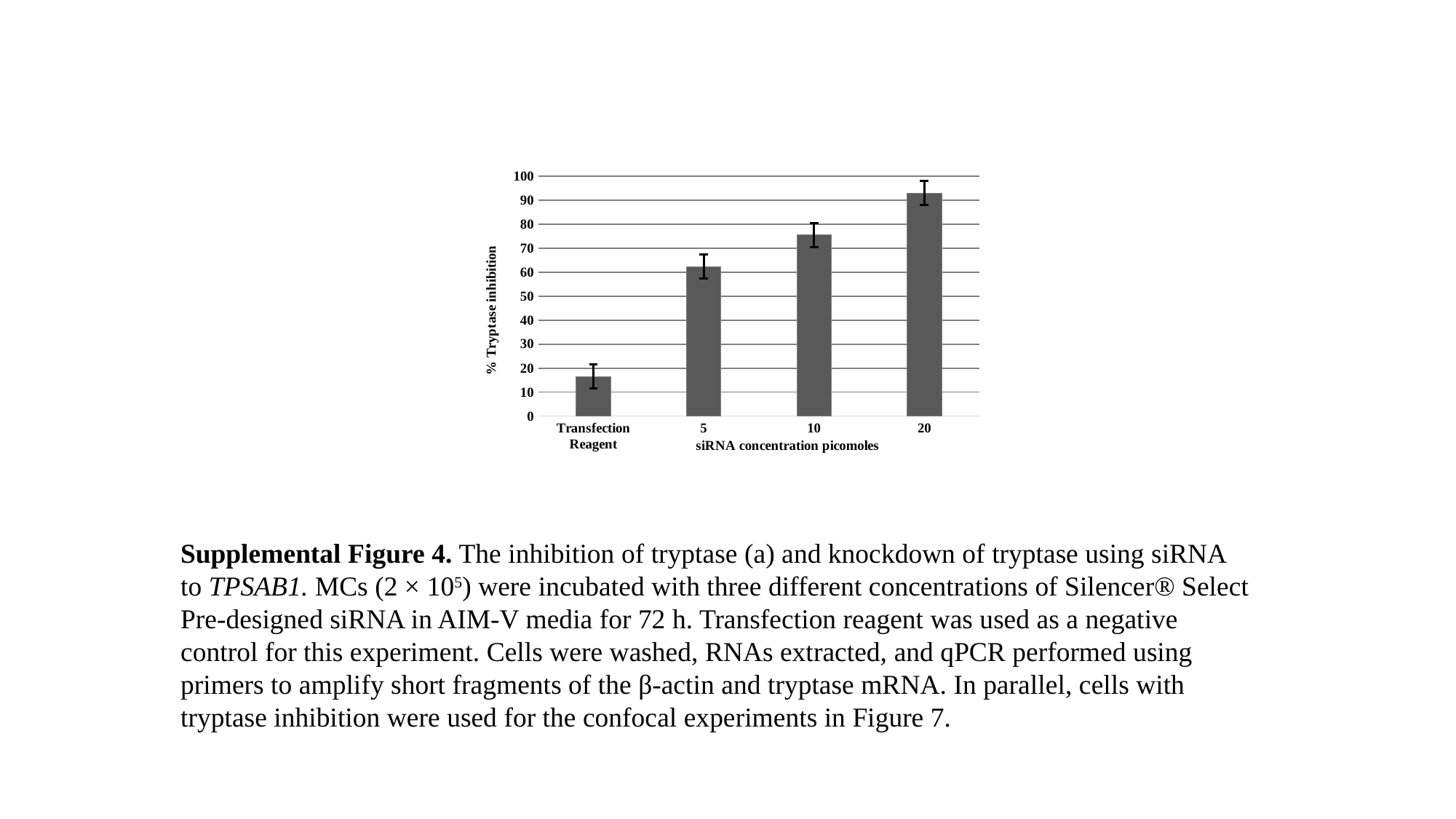

### Chart
| Category | |
|---|---|
| Transfection Reagent | 16.497418961534837 |
| 5 | 62.365768317370986 |
| 10 | 75.4638982397279 |
| 20 | 92.94778744956517 |Supplemental Figure 4. The inhibition of tryptase (a) and knockdown of tryptase using siRNA to TPSAB1. MCs (2 × 10^5^) were incubated with three different concentrations of Silencer® Select Pre-designed siRNA in AIM-V media for 72 h. Transfection reagent was used as a negative control for this experiment. Cells were washed, RNAs extracted, and qPCR performed using primers to amplify short fragments of the β-actin and tryptase mRNA. In parallel, cells with tryptase inhibition were used for the confocal experiments in Figure 7.
